# The 1257 Samalas eruption (Lombok, Indonesia): the single greatest stratospheric gas release of the Common Era

**DOI:** 10.1038/srep34868

**Published:** 2016-10-10

**Authors:** Céline M. Vidal, Nicole Métrich, Jean-Christophe Komorowski, Indyo Pratomo, Agnès Michel, Nugraha Kartadinata, Vincent Robert, Franck Lavigne

**Affiliations:** 1 Institut de Physique du Globe de Paris, Université Sorbonne Paris Cité, CNRS UMR 7154, Paris, 75005, France; 2 Museum of Geologi, Badan Geologi, Bandung, 40122, Indonesia; 3 Center of Volcanology and Geological Hazards Mitigation, Badan Geologi, Bandung, 40122, Indonesia; 4 Observatoire Volcanologique et Sismologique de la Guadeloupe IPGP, Gourbeyre, 97113, France; 5 Laboratoire de Géographie Physique UMR 8591, Université Paris 1 Panthéon-Sorbonne, 92195 Meudon, France

## Abstract

Large explosive eruptions inject volcanic gases and fine ash to stratospheric altitudes, contributing to global cooling at the Earth’s surface and occasionally to ozone depletion. The modelling of the climate response to these strong injections of volatiles commonly relies on ice-core records of volcanic sulphate aerosols. Here we use an independent geochemical approach which demonstrates that the great 1257 eruption of Samalas (Lombok, Indonesia) released enough sulphur and halogen gases into the stratosphere to produce the reported global cooling during the second half of the 13th century, as well as potential substantial ozone destruction. Major, trace and volatile element compositions of eruptive products recording the magmatic differentiation processes leading to the 1257 eruption indicate that Mt Samalas released 158 ± 12 Tg of sulphur dioxide, 227 ± 18 Tg of chlorine and a maximum of 1.3 ± 0.3 Tg of bromine. These emissions stand as the greatest volcanogenic gas injection of the Common Era. Our findings not only provide robust constraints for the modelling of the combined impact of sulphur and halogens on stratosphere chemistry of the largest eruption of the last millennium, but also develop a methodology to better quantify the degassing budgets of explosive eruptions of all magnitudes.

Sulphur (S) gases injected into the stratosphere during plinian eruptions are converted into sulphate aerosols that travel around the globe and backscatter solar radiation, resulting in a net cooling of the troposphere and the Earth’s surface^1^. Besides the effect of sulphate aerosols, volcanogenic halogens and especially chlorine (Cl) and bromine (Br) may induce the catalytic destruction of the ozone layer^2^. Although these processes have been observed on a very small scale for a number of recent explosive eruptions such as the 1963 Agung, 1982 El Chichón and 1991 Pinatubo events^3^, the effects of large historic silicic eruptions on climate and atmospheric chemistry remain poorly constrained, with hitherto unexplored feedbacks. The reconstruction of climate forcing associated with explosive eruptions involves several scientific disciplines, as it strongly depends on a variety of parameters including the height of injection, the gas flux and the latitude of the volcanic source. For past plinian eruptions suspected to have triggered or enhanced climate cooling, temperature reconstructions are commonly based on the amount of associated sulphate emissions inferred from glaciological records. It is furthermore commonly admitted that the presence of a pre-eruptive vapour phase renders the quantification of the emissions of sulphate and other volatile species of past eruptions particularly challenging^4^,^5^,^6^.

The 1257 eruption of Mt Samalas, a part of the Rinjani volcanic complex (Fig. 1) on Lombok Island (Indonesia), has been recognized as the “mystery eruption”^7^ associated with the largest sulphate spike of the last 2.3 ky recorded in cores from both Arctic and Antarctic ice sheets^8^. This continuous four-phase eruption evacuated 40 ± 3 km^3^ of trachydacitic magma during tens of hours, producing plinian plumes that rose up to 43 km in the stratosphere and tephra fingerprinted up to 660 km from the source, thus standing as the most powerful eruption of the last millenium^9^. Archaeologists recently determined a date of 1258 for mass burial of thousands of medieval skeletons in London^10^, that could be linked in some respect to climatic perturbations in the Northern Hemisphere by the 1257 Samalas eruption. Indeed, medieval chronicles in Northern Europe^7^ document the occurrence of initial warming in the early winter of 1258 just following the eruption, that was followed by extensive wet and cold climatic conditions in 1259 that may have impacted crops and contributed to the onset and magnitude of famines at that time for some regions of the Northern Hemisphere. The 1257 Samalas eruption might also have contributed to the onset of the Little Ice Age^11^.

The climate-forcing associated with the 1258–1259 sulphate anomaly in ice-cores has been the subject of intense research^12^,^13^,^14^,^15^, suggesting a range of sulphate yields until the source of the eruption was found. The recent re-evaluation of the record of volcanic sulphate deposition based on an extensive array of Antarctic ice cores suggested that a total of 170 Mt of SO_2_ (85 Tg of S) would have been released to produce the observed sulphate spikes, greater than the 45 Mt of SO_2_ estimated for the 1815 Tambora eruption^16^. Temperature reconstructions based on tree-ring proxies and ice-core records showed that the emission of 96 to 138 Tg of SO_2_, the most probable scenarios, would have induced an extra-tropical summer cooling over land of −0.6 °C to −5.6 °C during a period of 4–5 years^17^. The discrepancies between these estimates derived from ice-cores data and modelling reflect the complexity of climate reconstructions based on hypothetical S yields based on distal proxies.

The quantification of volcanogenic volatile emissions at the source commonly relies on the residual amount of volatiles dissolved in silicate melts prior to eruption recorded in small droplets of magma trapped in crystals (melt inclusions) of the erupted products^18^. Here we propose an alternative approach based on melt inclusions recording the different steps of magma evolution, including melt inclusions representative of both the magma that fed the eruption (a three-phased mixture of melt, gas and crystals) and of the residual melt.

## Pre-eruptive magmatic conditions

The detailed petrology and mineralogy of the Holocene lavas and scoria fallouts of the Rinjani volcanic complex, including the magmatic processes leading to the 1257 Samalas eruption, are presented by Métrich *et al.* (in review), and are briefly summarised hereafter. The 1257 eruption evacuated a chemically homogeneous magma body of trachydacite (64.0 ± 0.4 wt% of SiO_2_; 8.1 ± 0.1 wt% of Na_2_O + K_2_O, volatile free) that derived from a parent high alumina basalt magma through fractional crystallization. The trachydacitic magma crystallised and degassed at temperatures of 900 °C to 980 °C. Its mineral paragenesis typically consists of plagioclase showing a bi-modal distribution with patchy zoned cores (An_82–75_) surrounded by bands (An_50–46_), in association with amphibole (magnesio-hastingsite), orthopyroxene (clinoenstatite), rare clinopyroxene (augite), titano-magnetite, iron sulphide and apatite.

**Figure 2:**
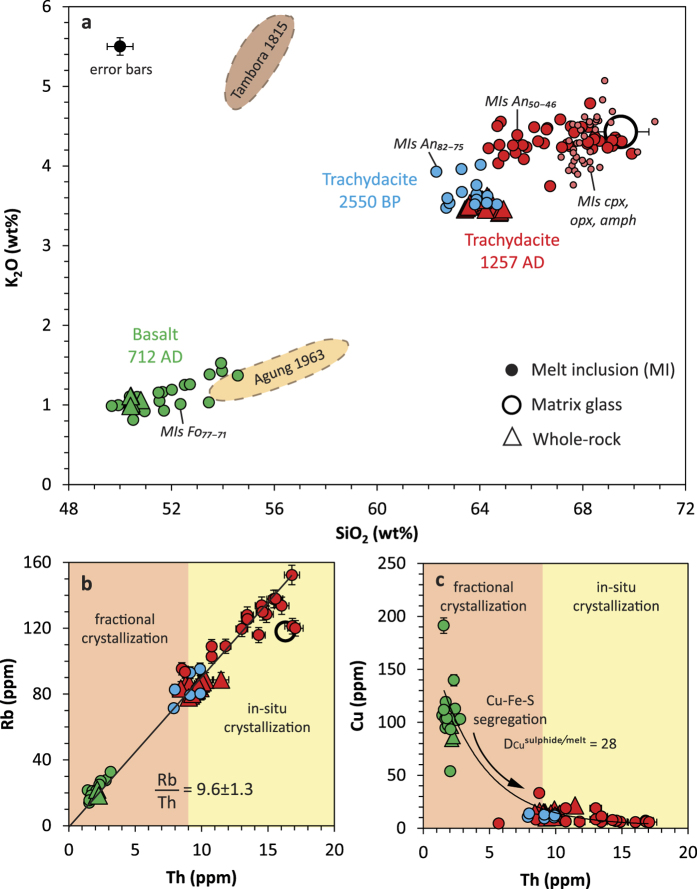
Melt inclusions record the magma evolution leading to the 1257 eruption. (**a**) K_2_O vs SiO_2_ variation diagram for the Rinjani-Samalas calc-alkaline suite including whole-rocks, melt inclusions in olivine of the 712 high alumina basalt (Fo is olivine forsterite content, i.e. 100 × *Mg*/(*Mg* + *Fe*)), in plagioclase of 2550 B.P. and 1257 pumice clasts (An is anorthite content, i.e. 100 × *Ca*/(*Ca* + *Na*)), in clino-(cpx), ortho-(opx) pyroxene, amphibole, and matrix glasses of the 1257 eruptive products. Compositions are normalised to 100 wt%, free of volatiles. The Rinjani-Samalas suite plots in between the whole-rock compositional fields of 1815 Tambora^53^ and 1963 Agung^64^,^65^ products, highlighting the enrichment in K_2_O of magmas towards the East of the Lesser Sunda arc. (**b**) Rb-Th positive correlation indicates that the 1257 trachydacite derived from its parent basaltic magma through a dominant process of fractional crystallization. Plagioclase An_82–75_-hosted melt inclusions are representative of the 1257 whole magma composition, whereas plagioclase An_50–46_-hosted melt inclusions record its shallow depth *in*-*situ* crystallization. (**c**) Cu vs Th variation diagram showing strong Cu fractionation during magma differentiation recording the prevalent Cu-sulphide segregation. See Supplementary Figures S2 and S3 for more major and trace element variation diagrams.

**Figure 3:**
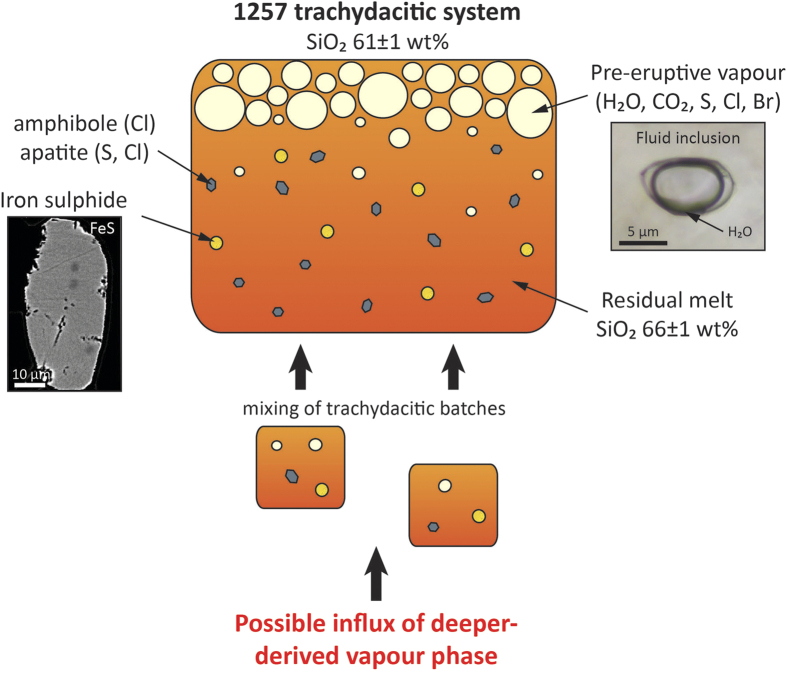
Volatile repositories in the 1257 trachydacitic system. Composition of the residual melt is recorded by melt inclusions trapped in cpx, opx and amphibole whereas that of the whole magma (melt + mineral phases + pre-eruptive vapour) is preserved by melt inclusions in plagioclase An_82–75_. The existence of pre-eruptive vapour is illustrated by the occurrence of water-rich fluid inclusions in minerals (Supplementary Figure S5). CO_2_ is very likely present in pre-eruptive vapour. The whole system is likely the result of the mixing of trachydacitic magma batches displaying distinct volatile contents (Supplementary Figure S4f).

Here we combined the detailed geochemical study of minerals and melt inclusions with matrix glass and whole-rock analyses (major, trace, S, Cl and Br) to characterise the composition of the pre- and post-eruptive magmatic system, and further quantify the volatile budget of the 1257 eruption. Samples from the four phases of the 1257 trachydacitic eruption and of the 712 A.D. basaltic scoria fallout have been examined (see Supplementary Sample description and Figure S1). Our dataset is complemented by melt inclusions from the trachydacitic pumice of the 2550 B.P. eruption^19^ (compositions are reported in Supplementary Tables S1, S2 and S3). Major and trace elements denote the bi-modality of the magma compositions and the lack of intermediate andesitic magma (Fig. 2a,b). The overall evolutionary trend is perfectly illustrated by the positive correlation between Rb and Th (Fig. 2b), suggesting that the 1257 trachydacite derived from the basalt through fractional crystallization of ~81% (Methods). Furthermore, compositions of olivine-hosted melt inclusions match that of the whole-rock parent basalt of the 1257 trachydacite (Fig. 2b). Most remarkable are the melt inclusions in plagioclase An_82–75_ that are representative of the whole trachydacitic magma (60.5 ± 1.3 wt% of SiO_2_; 9.0 ± 0.9 ppm of Th) whereas more sodic plagioclase (An_50–46_), amphibole and pyroxenes trapped melts that recorded the evolution towards the residual trachydacitic composition of the matrix glass (68 ± 1 wt% of SiO_2_; 16.3 ppm of Th) due to *in*-*situ* crystallization at shallow depth. Depicting the magma evolution respectively associated with fractional crystallization and *in*-*situ* crystallization is an innovative approach that enables to decipher the volatile evolution from the parent basalt to the 1257 trachydacite. We further quantify the distribution of sulphur and halogens between the pre-eruptive silicate melt (melt inclusions), the volatile-bearing mineral phases (sulphide (S), apatite (SO_2_, Cl) and amphibole (Cl)), and the vapour phase (Fig. 3) consisting of gas species exsolved during *in*-*situ* crystallization of the trachydacite in the shallow crust, with possible incremental influx of deeper-derived vapour phase^20^. Finally we establish the degassing budget of the 1257 Samalas eruption by comparing the pre-eruption and the post-eruption trachydacitic magma. We stress that volatile partitioning between the vapour phase and such sulphide-saturated calc-alkaline melts at 900–980 °C is still poorly constrained by experimental data.

**Figure 4:**
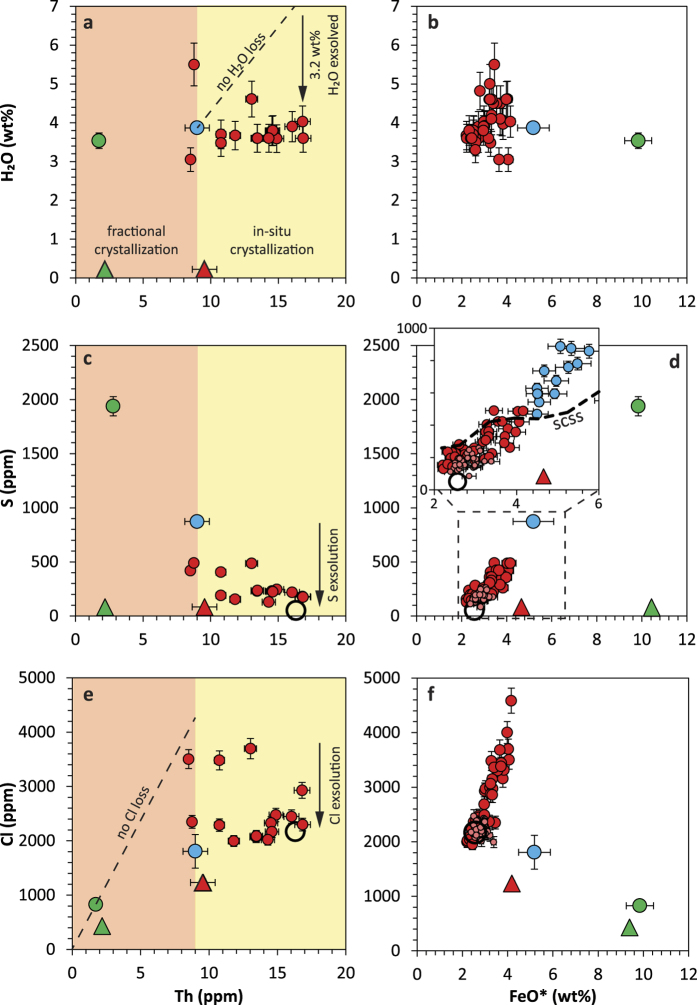
Melt inclusions record the volatile evolution through magma differentiation and *in*-*situ* crystallization. (**a**) H_2_O vs Th and (**b**) H_2_O vs FeO* contents in melt inclusions reflect water exsolution through magma differentiation. Reported average H_2_O content of basalt are calculated considering melt inclusions unaffected by H^+^ diffusion through host mineral (Supplementary Figure S6b). (**c**) S vs Th variation diagram in melt inclusions. (**d**) Positive correlation of S and FeO* in the 1257 melt inclusions is consistent with sulphide saturation. The S concentration at sulphide saturation (SCSS, dashed curve) was calculated with model B of Fortin *et al.*^25^. (**e**) Cl vs Th contents in melt inclusions and whole-rocks suggest that Cl has an incompatible behaviour during basalt differentiation. (**f**) Cl vs FeO* contents in melt inclusions, whole-rocks and matrix glasses reflect mixing between trachydacitic melts and Cl exsolution during *in*-*situ* crystallization of the 1257 trachydacite. The process of mixing is also demonstrated by the positive correlation of S and Cl (Supplementary Figure S4f). Symbols as in Fig. 2. Average initial volatile contents of melt inclusions representative of the basaltic magma and of the whole 1257 system as well as whole-rock compositions are reported with error bars (1*σ*).

The evolution of volatile species illustrated in Fig. 4 reflects the differential ability of each species to be fractionated into the vapour phase and minerals. Water contents of melt inclusions remain relatively constant along the path of magma differentiation (Fig. 4a,b). The basalt displays average water contents of 3.5 ± 0.2 wt%, a typical value for arc basalts^21^. The 1257 trachydacite magma derived from the basalt contains 3.9 ± 0.1 wt% of H_2_O, a value measured in melt inclusions in plagioclase An_82–75_. Such a dissolved amount of H_2_O is well below the maximum water concentrations (8–<10 wt%) of amphibole-bearing dacite crystallizing at 300–400 MPa under water-saturated conditions^22^. This suggests a drastic loss of water during fractional crystallization and most likely during magma decompression and ascent. The distribution of the H_2_O concentrations in melt inclusions in plagioclase An_50–46_ indicates that ~70% cluster at 3.7 ± 0.3 wt% H_2_O (Supplementary Figure S4a), a value representative of the H_2_O concentration of the residual melt (Fig. 4b). A few higher water contents (up to 5.5 wt%) reflect the multi-stage exsolution of water from the melt, and possibly magma mixing during refilling of the 1257 trachydacitic system. This feature is consistent with plagioclase textures and the temperature gradient (900–980 °C) calculated from thermometers (Métrich *et al.*, in review). Fluid inclusions fully support the presence of a free water-rich vapour phase (Supplementary Figure S5a,b). No dissolved or exsolved CO_2_ was detected, even though some fluid inclusions show evidence of carbon bearing-complexes (Supplementary Figure S5e). Considering that the trachydacitic system contains an initial amount of 3.9 ± 0.1 wt% of dissolved H_2_O, we estimate a proportion of exsolved water of 3.5 wt% during *in*-*situ* crystallization of the trachydacite (Fig. 4a). The water content of the residual melt corresponds to a fluid pressure 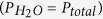 of 90–120 MPa under equilibrium conditions, suggesting an upper limit of magma storage of 3.3–4.4 km assuming a crustal density of 2.8 g/cm^3 ^23. Hence, the 1257 Samalas eruption was sustained by a trachydacite reservoir developed in the upper crust similarly to other large plinian eruptions^24^.

Sulphur concentrations in melt inclusions reflect a more complex behaviour (Fig. 4c,d). The parent basalt displays an average S content of 1940 ± 90 ppm, a value commonly measured in moderately oxidized, hydrous, arc basalts^20^. Poly-metallic sulphides (Cu-Fe-S) partitioning Cu (Fig. 2c) do not occur in S-rich melt inclusions but are ubiquitous in partly degassed glass embayments. The trachydacitic system displays a total S content of 870 ± 20 ppm distributed between the melt, the S-bearing minerals and the free gas phase. Sulphur concentrations in melt inclusions of clinopyroxene (150 ± 20 ppm), orthopyroxene (180 ± 45 ppm) and amphibole (185 ± 45 ppm) are similar to that of the most evolved plagioclase-hosted melt inclusions (Fig. 4d). Matrix glasses are the most degassed endmember with 50 ± 20 ppm of S (Supplementary Table S2). The positive correlation of S and FeO (Fig. 4d) corresponds to the S concentration at sulfide saturation (SCSS)^25^, in agreement with the occurrence of iron sulphide globules (up to 60 *μm* in size) entrapped with apatite in phenocrysts (mostly amphibole). However, the extent of FeO content range reflects the possible effect of magma mixing between melts displaying distinct S contents. The composition of iron sulphides (Supplementary Table S4) enabled to estimate a proportion of 0.01% of Fe-sulphides in the system (Methods), suggesting that they removed a negligible amount of S (~50 ppm) from the melt. Furthermore, the range of S concentrations in apatite (0.01–0.26 wt% of S, Supplementary Figure S6) indicates the sporadic influx of SO_2_ in the gas phase^26^, as supported by the occurrence of submicroscopic crystals of anhydrite and gypsum (and other S-bearing complex minerals) on the walls of fluid inclusions (Supplementary Figure S5c–e).

The average Cl concentration in Rinjani-Samalas basalts (830 ± 80 ppm) is typical of a volcanic arc domain^27^. The trachydacitic system displays a total Cl content of 3500 ± 175 ppm (Fig. 4e) accounting for the Cl distributed between the melt, the Cl-bearing minerals (amphibole and apatite) and the pre-eruptive vapour. Simple mass balance calculation based on apatite composition (42.9 ± 0.2 wt% of P_2_O_5_ and 0.89 ± 0.02 wt% Cl; Supplementary Table S5) indicates that up to 1.5 ± 0.3% of apatite crystallised (Methods) with a negligible effect on the Cl concentration of the melt (~130 ppm). Furthermore, amphibole from the 1257 trachydacite with an average Cl content of 700 ± 100 ppm (Supplementary Table S6) only removed a minor amount of Cl from the melt. Residual melts trapped in pyroxenes (2250 ± 140 ppm), and amphibole (2170 ± 210 ppm) share similar Cl concentrations with matrix glasses (2170 ± 210 ppm; Supplementary Table S2), indicating low Cl exsolution from the melt during eruption. Cl is therefore dominantly exsolved into the pre-eruptive vapour phase at shallow depth, a feature that explains the significant decrease of the Cl/Th ratio during *in*-*situ* crystallization of the trachydacitic magma (Fig. 4e). Furthermore, the positive correlation between Cl and FeO (Fig. 4f) corroborates mixing of partly degassed trachydacitic magma batches differing by their volatile contents.

Pumice clasts from the four phases of the eruption contain on average 1230 ± 45 ppm of Cl (Table 1). This value is in agreement with the residual Cl content of matrix glasses after correction for crystallization. Br contents average 1.6 ± 0.2 ppm in basaltic scoria and 2.92 ± 0.08 ppm in 1257 pumice clasts (Table 1). The average Br/Cl ratio of the basaltic whole-rock (3.7 ± 0.4 × 10^−3^) is in agreement with the upper range of published values for arc/back-arc basin basalts (1–3.2 × 10^−3^)^28^. This probably suggests a minimum effect of syn-eruptive degassing on the Br/Cl ratio^29^. Considering that the whole-rock Br/Cl ratio (3.7 ± 0.4 × 10^−3^) is characteristic of the basaltic magma ratio, we calculated indirectly a Br concentration of 3.1 ± 0.4 ppm in the basalt containing 830 ± 80 ppm of Cl (Table 1). Such a Br content is comparable to the concentrations measured in basaltic melt inclusions of Masaya, Nicaragua^30^. We stress that from the onset to the end of the 1257 eruption, whole-rocks record similar volatile concentrations (S, Cl, Br), suggesting a relatively constant extent of the syn-eruptive degassing.

## Atmospheric volatile emissions of the 1257 Samalas eruption

The atmospheric volatile yields from volcanic eruptions are usually estimated using the *petrologic method*, which involves substracting the volatile concentrations in the residual melt corrected for crystallization from those of undegassed melt inclusions^31^. The pre-eruptive volatile contents of the residual melt (170 ± 50 ppm of S and 2235 ± 155 ppm of Cl) and that of the degassed matrix glass (50 ± 20 ppm of S and 2170 ± 110 ppm of Cl) indicate volatile losses of 140 ± 50 ppm of S and 960 ± 190 ppm of Cl (Table 1). When scaled to the mass of erupted magma (calculated using a volume of 40 km^3^ and a DRE density of 2500 kg/m^3^, see Methods), the syn-eruptive devolatilisation of the melt would have released 14 ± 5 Tg of S (28 Tg of SO_2_) and 96 ± 19 Tg of Cl. This calculation neglects, however, the amounts of S and Cl stored in the pre-eruptive vapour phase, and thus provides minimum estimates.

In the following, we quantify the atmospheric yields using the representative volatile contents of the whole 1257 trachydacitic system. Its S content is recorded by melt inclusions trapped in plagioclase An_82–75_ (Table 1) and accounts for the S allocated in the trachydacitic melt, the pre-eruptive vapour phase, and the iron sulphides (Fig. 3). We further assume that sulphide breakdown prior to eruption through interaction with a pre-existing fluid phase^32^ did not occur, as they likely remained stable prior to eruption. Given the negligible effect of iron sulphide on the S dissolved in the melt, the S degassing (melt + vapour) is provided by the difference between the initial S concentration of the system (870 ± 20 ppm) and the S content of the 1257 whole-rock (80 ± 20 ppm), which yields a loss of 790 ± 30 ppm (Table 1). Such a loss corresponds to the emission of 79 ± 6 Tg of S (158 ± 12 Tg of SO_2_).

The highest Cl content (3500 ± 175 ppm) recorded by the trachydacitic melt inclusions representative of the 1257 whole-rocks (Fig. 4e) accounts for the Cl allocated in the melt and in the pre-eruptive vapour phase. Using the average Cl concentration in the whole-rock (1230 ± 45 ppm), we calculate a Cl loss of 2270 ± 180 ppm, indicative of an atmospheric discharge of 227 ± 18 Tg of Cl (Table 1).

Underplating basalt may contribute to volatile emissions during plinian eruptions of silicic magmas^6^. Despite the absence of any obvious petrologic evidence of the contribution of the parent basalt into the degassing budget, we further explore how the potential contribution of a deeper-derived vapour phase would have increased the atmospheric emissions of the 1257 eruption. Using the S concentration of basaltic melt inclusions, we calculate that the trachydacitic system derived through 81% of fractional crystallization of the parent basalt would have contained 1 wt% of S (*S*^*system*^; Table 1), distributed between the trachydacitic melt (*S*^*melt*^), the pre-eruptive vapour phase (*S*^*fluid*^), and the poly-metallic sulphides (*S*^*Cu*–*Fe*–*S*^). The Cu concentration decreases from basalt to trachydacite (Fig. 2c) indicating that segregated Cu-bearing sulphides (Cu–Fe–S) sequestered a maximum of 690 ppm of S (Methods; Table 1). Considering such a sink of S in Cu-sulphides and the proportion of S dissolved in the trachydacitic melt (870 ± 20 ppm), the pre-eruptive vapour phase would have stored a maximum of 8500 ppm of S. This suggest a partition coefficient 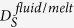 of 13, which fills a gap of experimental constraints on S solubility for these magma conditions. In order to calculate the mass of S that could have been emitted by additional pre-eruptive vapour, it is necessary to assess the amount of water lost during the magma differentiation. An amount of 5–6 wt% fluid is thought to be an upper limit beyond which percolation would occur and the fluid would be lost from the system^33^. Furthermore, an average value of 5 wt% fluid is consistent with the discrepancy between the water content of the trachydacitic melt (3.7 ± 0.3 wt%) and the maximum H_2_O concentrations that an amphibole-bearing dacite could display (8–<10 wt%)^22^. Assuming a maximum of 5 wt% of fluid in the system including 3.5 wt% exsolved during *in*-*situ* crystallization of the trachydacite, the parent basalt would have contributed to 1.5 wt% of supplementary vapour. This corresponds to an addition of 13 ± 6 Tg of S onto the S budget (Table 1). The large uncertainty on this estimate reflects the complexity of quantifying the proportion of water, the major component of the vapour phase. The 1257 eruption would thus have released a maximum of 92 ± 8 Tg of S (184 ± 16 Tg of SO_2_).

We estimate similarly a total theoretical Cl available in the trachydacitic system of 4300 ± 720 ppm (Table 1). Within the error, such concentration is relatively in agreement with the initial Cl content of the melt inclusions representative of the 1257 whole-rocks (3500 ± 175 ppm), suggesting minor Cl fractionation by Cl-bearing phases during basalt differentiation. The potential contribution of the parent basalt onto the Cl budget is thus likely negligible.

Partial exsolution of magmatic Br into pre-eruptive vapour may occur, and its fluid/melt partition coefficient 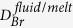 is typically much larger than 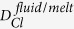 34. Following the previous reasoning, the initial Br concentration calculated for the parent basalt indicates a total of 16 ± 3 ppm Br available in the trachydacitic system (Table 1) allocated in both the melt and the pre-eruptive vapour phase. Using the average Br content of the 1257 whole-rocks (2.92 ± 0.08 ppm), we calculate a Br loss of 13 ± 3 ppm that corresponds to a maximum emission of 1.3 ± 0.3 Tg of Br (Table 1).

## The largest volatile release of the Common Era

**Figure 5:**
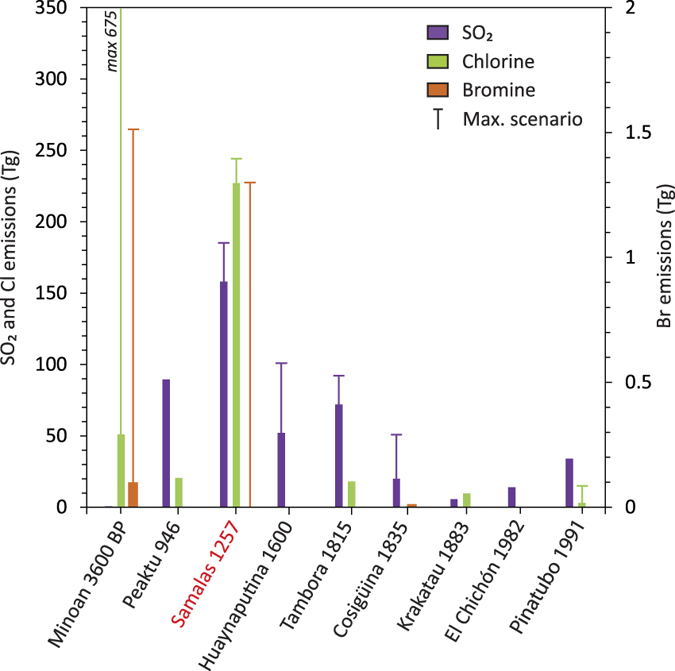
The 1257 Samalas eruption produced the largest volatile emissions of the Common Era. Plots of SO_2_, Cl and Br emissions (in Tg, i.e. megatons) for climate-impacting plinian eruptions. Yields of eruptions before 1980 are from petrological studies. Fine lines represent the maximum estimates (associated with the contribution of the parent basaltic magma in the case of the 1257 eruption). References: Minoan 3,600 y B.P. (Greece)^37^; Peaktu 946 A.D. (DPRK/China)^36^; Huaynaputina 1600 (Peru)^66^; Tambora 1815 (Indonesia), emissions re-calculated at 36–45 Tg S (73–91 Tg SO_2_) and 18–23 Tg Cl based on the syn-eruptive losses (400 ppm S; 200 ppm Cl)^53^,^67^ and new volume estimates of 41 ± 4 km^3^ DRE^68^ and 51 km^3^ DRE^69^); Cosigüina 1835 (Nicaragua)^30^,^70^; Krakatau 1883 (Indonesia)^71^; El Chichón 1982 (Mexico)^72^; Pinatubo 1991 (Philippines)^4^.

The 1257 Samalas eruption produced a S injection of 79 ± 6 Tg, equivalent to 158 ± 12 Tg of SO_2_ (Table 1), and a maximum of 184 ± 16 Tg of SO_2_ if the parent basalt contributed to the degassing budget. Hence, the influx of volatiles from the basalt would not exceed 15% of the total budget. Furthermore, our new approach increases by a factor of 5 the minimum S emission derived from the classic petrologic method. We stress that such emission probably occurred within a day^9^, and is much higher than the total annual global volcanic SO_2_ flux of 15–21 Tg/yr associated with atmospheric emissions from both quiescent and explosive degassing volcanoes^35^. In addition to the prodigious S yield, the 1257 Samalas eruption emitted 227 ± 18 Tg of Cl, and a maximum of 1.3 ± 0.3 Tg of Br taking into account the potential contribution of the basalt. These yields stand as the largest volatile emissions of the Common Era (Fig. 5), exceeding by a factor of two the SO_2_ emissions of the 946 Peaktu eruption (90 Tg of SO_2_)^36^, and of the 1815 Tambora eruption (73–91 Tg of SO_2_) that had a devastating impact on climate on a global scale. Furthermore, the Cl yield of the Samalas eruption constitutes the largest emission since the Minoan eruption of Santorini, 3,600 y B.P.^37^. Although the Br emissions have been estimated for only a few Holocene eruptions, the Br release of the 1257 Samalas eruption (1.3 ± 0.3 Tg) is similar to the maximum scenario of Br yield of the Minoan eruption (1.5 Tg of Br)^37^, and one order of magnitude higher than the Br emission of the Chiltepe eruption, 1.8 ka (0.125 Tg)^30^.

## Stratospheric injection and potential impact on climate and atmospheric chemistry

Sulphur gases are scavenged by incorporation into ice particles in volcanic plumes before reaching the tropopause, resulting in stratospheric emissions which probably represents 80% of the initial injection^38^. Such a process would suggest that the 1257 Samalas eruption produced a stratospheric injection of ~126 Tg of SO_2_ (Table 1), which is within the range of the two SO_2_ stratospheric scenarios of 96 and 138 Tg of SO_2_ estimated based on climate modelling and tree-ring based hemispheric temperature reconstructions^17^. If the parent magma contributed to the degassing budget, the stratospheric injection of SO_2_ could reach up to 150 Tg, which should be considered as a maximum value. Hence, our estimates corroborate the upper range of sulphate yields derived from ice-core data and temperature reconstructions. We also stress that the low discrepancies between our stratospheric estimates and ice-cores based scenarios may reflect the efficient gas injection from the onset to the end of the eruption. Indeed, the stratospheric loading of volatiles is correlated to the initial dynamics of an eruption that needs to be sufficiently powerful to inject a pre-existing vapour phase accumulated at the top of the magmatic reservoir^33^,^39^ into the stratosphere via a high-flux gas jet and convective column before wholesale column collapse.

The input of both volcanic Br and Cl enhances the catalytic destruction of ozone because the resulting BrO is a reaction partner for ClO^40^. Such reactions can occur either when halogens are released at tropospheric altitudes or when they are directly injected into the stratosphere. The Cl and Br emissions of the 1257 Samalas eruption represent initial atmospheric concentrations of 36 ppbv of Cl and 90 pptv of Br (Table 1). For comparison, the pre-industrial atmospheric Cl mixing ratio was 0.55 ppbv, corresponding to methyl chloride emissions from the oceans, i.e. before the onset of anthropogenically dominated emissions, and increased up to 3.8 ppbv in the late 1990s, during the Antarctic ozone hole climax^41^,^42^. The pre-industrial Br mixing ratio was 5 pptv, and increased up to 20 pptv in the late 1990s^43^. Hence, the 1257 Samalas eruption produced increments in globally averaged Cl and Br concentrations that exceed the pre-1980 levels by a factor of 65 for Cl and 18 for Br, and the ozone-hole climax concentrations by factors of 9 for Cl and 5 for Br. These results likely suggest that the 1257 eruption discharged enough halogen gases into the atmosphere to trigger ozone destruction cycles. We stress that such comparisons should be made carefully, however, given that the mixing ratios calculated for the 1257 Samalas eruption correspond to initial and local increases of Cl and Br concentrations, whereas background mixing ratios are global annual values.

A significant proportion of Cl and Br released by the 1257 Samalas eruption was likely scavenged by hydrometeors during ascent in the plume and may not have reached the stratosphere. Although sattelite-based measurements of present-day volcanism enhanced our understanding of the fate of volcanic halogens in the stratosphere, much remains to be investigated, in particular concerning Br emissions and potential effects of large tropical explosive eruptions on stratospheric ozone^44^. Sophisticated plume experiments and modelling^38^ showed that 10% to 25% of halogen gases can reach the stratosphere, although the scavenging efficiency strongly depends on several parameters such as the latitude, the salinity of the fluid, the gas phase composition and the ability of ash particles to capture halogens^45^. Comparison of tropical versus high latitude eruptions shows that tropical humid atmospheric conditions, including the occurrence of a typhoon, may cause efficient scavenging of halogens as observed for the 1991 Pinatubo eruption^46^,^47^. It has been observed in the case of the 1982 El Chichón eruption, however, that a high mass flux rate within a plinian plume can partly preserve halogens from being scavenged, thus enhancing the stratospheric injection^48^. Considering that: (i) the 1257 Samalas eruption more likely occurred between May and July 1257, i.e. during the Indonesian dry season^7^,^17^; and (ii) the strong mass flux rates of the two plinian phases (2.8 × 10^8^ kg/s during phase 1 and 4.6 × 10^8^ kg/s during phase 3)^9^ are one order of magnitude higher than the maximum flux rate of the 1982 El Chichón eruption (6.8 × 10^7^ kg/s)^49^,^50^, we assume that the stratospheric injection of halogens could likely have reached 10 to 25% of the initial load. This would correspond to stratospheric injections of 23–57 Tg of Cl and 0.1–0.3 Tg of Br (Table 1) for the 1257 eruption. Volcanic-induced ozone depletion has been observed as a consequence of the 1991 Pinatubo eruption that caused the destruction of 2–6% of global average total ozone^46^ due to the release of 3–16 Tg of Cl^4^ most of which was scavenged by typhoon Yunya during the eruption. The combined Cl (51–675 Tg) and Br (0.1–1.5 Tg) emissions of the Minoan eruption of Santorini (Greece) would have provoked reductions of 20 to >90% of ozone at northern high latitudes^37^. This strongly suggests that the 1257 Samalas eruption likely provoked strong ozone destruction, even for the lower-bound 10%-injection scenario. This process is enhanced by the simultaneous emission of S, as sulphate aerosols provide surface area for heterogeneous chemical reactions that activate Cl and Br species, thus enabling and enhancing the catalytic destruction of ozone^51^,^52^.

Our study underscores the fundamental importance of considering magmatic systems in their totality as well as the evolution of the behaviour of volatiles during magma differentiation in order to significantly improve the quantification of the degassing budget of large climate-impacting explosive eruptions. The application of this methodology to other magmatic systems that have produced volatile-rich intermediate magmas would enhance the reconstruction of the climate impact of past volatile emissions associated to eruptions of large to moderate volumes of S-rich magmas (e.g. 1815 Tambora^53^, 1835 Cosigüina^54^). Given the prodigious amounts of volatiles released by the 1257 Samalas eruption, interactions between processes involving S, Cl and Br should be considered in future global scale climate-modelling. Although the probability of occurrence of such a large eruption in the next decades is statistically low, the ozone-destruction power of volcanic eruptions in general should be systematically assessed, particularly given that the Antarctic ozone-hole is decreasing^43^ and that stratospheric halogen concentrations are expected to reach their pre-industrial level during the second half of the century.

## Methods

### Calculation details

#### Fraction of solid removal

We apply Rayleigh’s law to Th concentrations (Th is highly incompatible) in basaltic and trachydacitic melts as 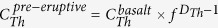, where 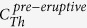 is the Th content of the 1257 system (9.0 ± 0.9 ppm), 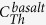 is the Th content of the basalt (1.7 ± 0.2 ppm), *f* is the proportion of remaining melt, and the global partition coefficient of Th *D*_*Th*_ is 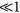. Equation is then simplified as 
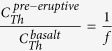
. We calculate a proportion of trachydacitic melt *f* of 0.19 ± 0.03, i.e. a solid removal (1−*f*) of 0.81.

#### Water loss during *in*-*situ* crystallization of the 1257 trachydacite

The conservation of the ratio of water respective to Th during *in*-*situ* crystallization of the trachydacite is expressed as 
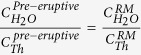
. *In*-*situ* crystallization of the 1257 trachydacite with 3.9 ± 0.1 wt% H_2_O and 9.0 ± 0.9 ppm Th indicates a theoretical water content of 7.2 wt% in the residual melt (RM). The latter actually contains 3.7 ± 0.3 wt% H_2_O, suggesting the exsolution of 3.5 wt% of H_2_O.

#### Proportion of apatite

Using P_2_O_5_ concentrations of the 1257 trachydacite (0.42 ± 0.05 wt%; Supplementary Table S3) and of the 1257 matrix glass (0.13 ± 0.06 wt%; Supplementary Table S3), we estimate a P_2_O_5_ loss Δ*P*_2_*O*_5_ of 0.6 ± 0.1 wt% due to apatite crystallization during *in*-*situ* crystallization. The proportion of P_2_O_5_ in apatite 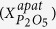 is given by 
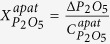
, where 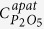 is the P_2_O_5_ content of apatite (42.9 ± 0.2 wt%; Supplementary Table S5). We thus calculate a proportion of 0.015 ± 0.003 of apatite that would account for the removal of a maximum of 130 ppm of Cl from the melt.

#### Proportion of Fe-sulphides in the 1257 trachydacitic system

The proportion of iron-sulphide in the 1257 trachydacite is derived from 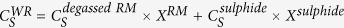, where *X*^*RM*^ is the residual melt fraction (0.59), and 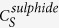 is the S content of Fe-sulphides (38.9 ± 0.5 wt%; Supplementary Table S4). This equation yields a proportion of iron-sulphides *X*^*sulphide*^ of 0.01% in the 1257 trachydacite.

#### Mass of volatile released

The mass of volatile *i* released is calculated as *m*_*i*_ = *m*_*magma*_ × Δ*i* × 10^−15^, where 10^−15^ is the conversion factor of ppm into kg, and *m*_*magma*_ = *ρ*_*m*_ × *V* is 1.00 ± 0.08 × 10^14^ kg (DRE magma density *ρ*_*m*_ is 2500 kg/m^3^, DRE erupted volume *V* is 40 ± 3 km^3 ^7).

#### Sulphur uptake by poly-metallic sulphides

Using Cu concentrations of the basalt (114 ± 32 ppm; Supplementary Table S3) and of the 1257 trachydacite (11.4 ± 0.8 ppm; Supplementary Table S3) derived through 81% of crystallization, we calculate that 580 ppm of Cu (Δ*Cu*) were fractionated by Cu-bearing sulphides segregated during the basalt differentiation. The amount of associated S is given by 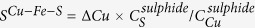 where 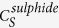 (32.1 ± 0.6 wt%) and 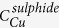 (27.0 ± 1.1 wt%) are S and Cu contents in Cu-Fe sulphides, respectively (Supplementary Table S4). This calculation yields an amount of 690 ± 225 ppm of S removed from the melt by poly-metallic sulphides. This amount is considered as a maximum because we neglected the possible fractionation of Cu into the vapour phase.

### Analysis of melt inclusions, matrix glasses and fluid inclusions

#### Water and CO_2_ analysis

Melt inclusions were analysed using a Thermo-Nicolet 6700 FTIR spectrometer coupled with an optical/IR Spectra-Tech microscope (IPGP, France). Water and carbon concentrations were calculated using the Beer-Lambert law (*C*(*wt*%) = 100 × *A* × *M*/(*ε* × *ρ* × *e*), where *A* is the absorbance, *M* the molar mass (g/mol), *ε* the molar absorptivity (L/mol · cm), *e* the thickness (cm), and *ρ* the glass density in g/L). Melt inclusion densities were calculated with the model of Lange and Carmichael (1987), using a partial molar volume of water of 12.0 ± 0.5 cm^3^/mol^55^. Dissolved total water concentrations (H_2_O_*molec*_ + OH^−^) were calculated using the broad absorption band at 3550 cm^−1^ and absorption coefficients of 62.8 L/mol · cm^56^ for basalt, and of 68.7 ± 1.0 L/mol · cm^57^ for trachydacite. Carbon concentrations were determined using the 1515 cm^−1^ 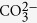 absorption band after subtraction of the background, previously acquired on a volatile-free basalt. Deconvolution of the carbonate peaks taking into account the contribution of the 1630 cm^−1^ H_2_O_*molecular*_ band was performed using PEAK FIT software. The absorption coefficient was calculated as *ε*_1515_ = 451 − 342 × *Na*/(*Na* + *Ca*)^58^. The analytical uncertainties on CO_2_ and water concentrations were 17% and ≥10%, respectively. CO_2_ or carbonate ions were not observed in trachydacitic melt inclusions.

#### Major element, S and Cl analysis

Major element concentrations in glasses and host minerals were measured by electron probe micro-analysis (EPMA) using a Cameca SXFive electron probe (Camparis, Paris, France). In melt inclusions they were measured with an accelerating voltage of 15 kV, a 4–10 nA defocused beam and peak counting times of 10–30 s depending on the element. Sodium was measured first with a 5 s peak count time in order to minimise alkali loss. S, Cl and P concentrations were determined with a 30 nA defocused beam and counting times of 120–200 s on peak. S speciation was investigated in these analytical conditions in a large olivine-hosted melt inclusion of the dataset, by scanning the peak position for the wavelength of S K*α* radiation (*λ*SK*α*)^59^. Accuracy of S and Cl measurements were ensured by analysis of KE12 (3332 ± 42 ppm Cl; 183 ± 6 ppm S; N = 57), of the alkali trachyte CFA47 (5399 ± 175 ppm Cl; 67 ± 17 ppm S; N = 61), and of the international standard Vg2 (291 ± 14 ppm Cl; 1435 ± 40 ppm S; N = 43). The analytical errors are ≤6% and 3% for S concentrations <200 ppm and above 1400 ppm, respectively; 4% and ≤2% for Cl concentrations <300 ppm and >3300 ppm, respectively. The detection limits were 55 ppm for S and 60 ppm for Cl.

#### Trace element analysis

Abundances of 30 traces elements (Ba, Ce, Co, Cu, Ni, Sc, V, Dy, Er, Eu, Gd, Hf, Ho, La, Lu, Nb, Nd, Pb, Pr, Rb, Sm, Sr, Ta, Tb, Th, Tm, U, Y, Yb and Zr) were determined in melt inclusions by laser ablation inductively coupled plasma mass spectrometry (LA-ICP-MS) at the Laboratoire Magmas et Volcans (Université Blaise Pascal, Clermont-Ferrand, France), using a 193 nm ArF excimer laser ablation system (Resonetics M50) coupled to a 7500 cs Agilent ICP-MS system, with helium as the ablation gas. Samples were analysed using a laser repetition rate of 2 Hz, laser spot diameter range of 20–40 mm and pulse energy of 6 mJ (14 J/cm^2^). The background was measured for 30 s before ablation, and each analysis lasted 100 s. Measurements were calibrated against NIST 612 glass standard^60^, using CaO as the internal element reference. Repeated analyses of BCR2-G glass international standard for each spot size were reproducible within 1 to ≤5% RSD for most elements and ≤7% RSD for Gd and some elements with low abundances such as Yb and Hf (<5 ppm) and Tb, Tm, Lu and Ta (<1 ppm). Our measurements on BCR-2G standard were compared to published values (Supplementary Table S7). Melt inclusions were analysed in single spot. Careful examination of counting statistics of each element and data reduction was performed using the Glitter software^61^.

#### Fluid inclusions characterisation

Plagioclase-hosted primary fluid inclusions of the 1257 A.D. rocks were analysed by Raman microspectrometry at the Ecole Normale Supérieure, Paris (France). Spectra were acquired in ambient conditions using a Renishaw INVIA spectrometer. This device is equipped with an Ar laser source giving an incident beam with a 514.5 nm wavelength, focused through a Leica microscope. The Rayleigh scattering component was removed by a Notch filter, and the Raman-scattered light was dispersed by a holographic grating with 1800 lines/mm and detected by a CCD camera. A power of 2 mW was used to avoid heating effects and sample damage. Raman spectra were acquired between 200 and 3900 cm^−1^ in order to identify S-, CO_2_- and H_2_O- bearing components of the fluid phase.

### Whole-rock analysis

Major and trace element analyses of whole-rock samples are reported in Métrich *et al.* (in review). In this work, S and halogens (Cl, F and Br) were extracted by the pyrohydrolysis method^62^. About 500 mg of powdered whole-rock mixed with ~500 mg of V_2_O_5_ in a platinum crucible was heated at 1200 °C in a quartz combustion tube through a H_2_O-vapour stream transported by a nitrogen flux. The extracted S and halogen species are converted into acids by hydrolysis in the H_2_O-vapour which was further condensed in a cooling system. The condensate was collected in a vial containing 10 mL of a NaOH solution (25 mmol/L). The vapour flux was adjusted in order to complete the extraction in 45–60 min and collect 80–100 ml of solution. The dilution factors (*mass*_*solution*_/*mass*_*sample*_) ranged from 180 to 250. After the complete extraction, the solutions were immediately analysed (for Cl and F) by liquid chromatography using a Dionex DX120 ion chromatograph (IC) with an Ion Pac AS9-HC (Dionex) anionic column, performed in suppression mode (ASRS-UltraII). The detection limit was 100 *μg*/L for both F and Cl in the sample solution. Solution concentrations were calculated using a calibration curve (0–50 ppm), and were further converted into rock concentrations using the dilution factor. Taking into account the mean dilution factor, detection limits in rock samples were 15 to 20 mg/kg for both elements. Br contents were determined using a 7900 Agilent ICP-MS device, in low resolution with a Scott spray chamber and a micro nebulizer (0.2 ml/min). Br was analysed in the ‘no gas’ mode, with an acquisition time of 0.3 sec, measuring 5 points as peak pattern, 3 replicates and 100 sweeps by replicates, 70 sec for the uptake, 40 sec as stabilisation time and 60 sec for rinsing time. Br solution concentrations were further calculated using a calibration curve (0–50 ppb), and were further converted into rock concentrations using the dilution factor. To ensure a complete extraction and the accuracy of the analyses, pyrohydrolysis/IC/ICP-MS was performed on international standards covering a wide compositional range and S, Cl, Br and F concentrations. We stress that our results were reproducible within 1–10% RSD for Cl, within 6–16% RSD for Br, and within 2–16% RSD for S except for AGV-1 (35% RSD). The efficient extraction of S from sulphide-bearing samples was guaranteed by the analysis of a sulfide-bearing syenite standard SY-2. Our results compared to published reference values are reported in Supplementary Table S8.

## Additional Information

**How to cite this article**: Vidal, C. M. *et al.* The 1257 Samalas eruption (Lombok, Indonesia): the single greatest stratospheric gas release of the Common Era. *Sci. Rep.* **6**, 34868; doi: 10.1038/srep34868 (2016).

## Supplementary Material

Supplementary Information

## Figures and Tables

**Figure 1:**
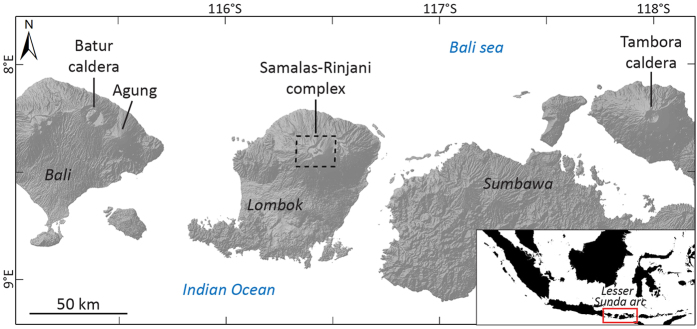
Map of the Lesser Sunda Islands and their active volcanoes. SRTM DEM at 3 arcsecond (~90 m) resolution (http://srtm.csi.cgiar.org)^63^ of Bali, Lombok and Sumbawa. This map was generated using the Esri ArcMapI 10.1 software (http:www.esri.com).

**Table 1:** Volatile degassing budget calculations.

	Th	S	Cl	Br
**Concentrations (ppm)**				
Undegassed residual melt *C*^*undegassed RM*^ (MIs cpx, opx, amph)		170 ± 50	2235 ± 155	
Degassed residual melt *C*^*degassed RM*^ (Matrix glass)	16.3	50 ± 20	2170 ± 110	
Pre-eruptive trachydacite *C*^*pre*–*eruptive*^ (MIs plagioclase ~An_76_)	9.0 ± 0.9	870 ± 20	3500 ± 175	
Trachydacitic whole-rock *C*^*WR*^	9.5 ± 0.9	80 ± 20	1230 ± 45	2.92 ± 0.08
Parent basalt *C*^*basalt*^ (MIs olivine ~Fo_76_)	1.7 ± 0.2	1940 ± 90	830 ± 80	3.1 ± 0.4
Basaltic whole-rock (712 AD scoria)	2.20 ± 0.07	39 ± 1	428	1.6 ± 0.2
**Classic petrologic method**				
Volatile *i* loss Δ*i* = *C*^*undegassed RM*^ − *C*^*degassed RM*^ × *X*^*RM*^ (ppm)		140 ± 50	960 ± 190	
Minimum mass released (Tg)		14 ± 5	96 ± 19	
**New approach: whole trachydacitic system**				
Volatile *i* loss Δ*i* = *C*^*pre*–*eruptive*^ − *C*^*WR*^ (ppm)		790 ± 30	2270 ± 180	
Mass released (Tg)		79 ± 6	227 ± 18	
**Contribution of the parent basalt**				
Theoretical amount of volatile available *C*^*system*^ (ppm)		10090 ± 1480	4300 ± 720	16 ± 3
*S*^*Cu*–*Fe*–*S*^ (ppm)		690 ± 225		
*S*^*fluid*^ (ppm)		8500 ± 1500		
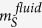 assuming 1.5 wt% fluid (Tg)		13 ± 6		
Volatile *i* loss Δ*i* = *C*^*system*^ − *C*^*WR*^ (ppm)				13 ± 3
Maximum mass released (Tg)		92 ± 8		1.3 ± 0.3
Global atmospheric mixing ratios (ppbv of Cl and pptv of Br)			36	90
Stratospheric emissions (Tg)		63–73	23–55	0.13–0.33

MIs: melt inclusions; cpx: clinopyroxene; opx: orthopyroxene; amph: amphibole.

Calculations of mass of volatile released and amount of S sequestered in Cu-Fe-S detailed in the method.

*X*^*RM*^ is the residual melt fraction, i.e. the ratio of Th contents in the 1257 trachydacitic whole-rock and the residual melt (0.59).

The concentration 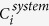 of each volatile species *i* in the 1257 trachydacitic system is derived from the 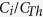 ratio of the parent basalt assuming the conservation of the ratio of volatile contents respective to Th during basalt differentiation.

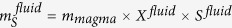
, where *X*^*fluid*^ is the maximum amount of deeper-derived vapour (1.5 wt%).

The maximum mass of S released is the sum of the emissions of the trachydacitic system (79 Tg S) and 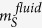 (13 Tg S). See text for Cl and Br.

Global atmospheric (troposphere and stratosphere) mixing ratios of halogen X is given by 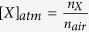, where *n*_*X*_ is the amount of substance (moles) of halogen X released by the eruption, and *n*_*air*_ is the amount of substance (moles) of air in the atmosphere, i.e. 1.5 × 10^20^ mol.

Stratospheric emissions calculated as 80% of S and 10–25% of Cl and Br total emissions^38^.

SO_2_ emissions (in Tg, i.e. megatons) correspond to twice the reported S emissions.
