# Supplementary material for: The 1257 Samalas eruption (Lombok, Indonesia): the single greatest stratospheric gas release of the Common Era
Source: Sci Rep. 2016 Oct 10;6:34868. doi: 10.1038/srep34868 (PMC5056521; doi:10.1038/srep34868)
Supplement: Supplementary Information [file srep34868-s1.pdf]

The 1257 Samalas eruption (Lombok, Indonesia): the single greatest stratospheric gas release of the Common Era

Céline M Vidal[1,*], Nicole Métrich[1], Jean-Christophe Komorowski[1], Indyo Pratomo[2], Agnès Michel[1], Nugraha Kartadinata[3], Vincent Robert[4], and Franck Lavigne[5]

[1]Institut de Physique du Globe de Paris, Université Sorbonne Paris Cité, CNRS UMR 7154, Paris, 75005, France
[2]Museum of Geologi, Badan Geologi, Bandung, 40122, Indonesia
[3]Center of Volcanology and Geological Hazards Mitigation, Badan Geologi, Bandung, 40122, Indonesia
[4]Observatoire Volcanologique et Sismologique de la Guadeloupe IPGP, Gourbeyre, 97113, France
[5]Laboratoire de Géographie Physique UMR 8591, Université Paris 1 Panthéon-Sorbonne, 1 Place Aristide Briand, 92195 Meudon, France, France
[*]vidal@ipgp.fr

Supplementary Information

Sample description and data treatment
Figure S1 Microphotographs of melt inclusions
Figure S2 Major element variation diagrams
Figure S3 Trace element variation diagrams
Figure S4 Volatile element variation diagrams
Figure S5 Fluid inclusions compositions
Table S1 Whole-rock compositions
Table S2 and S3 Melt inclusions compositions
Table S4 Composition of sulphides
Table S5 and Figure S6 Apatite compositions
Table S6 Composition of amphibole
Table S7 Standards for trace element analysis in melt inclusions
Table S8 Standards for volatile element analysis of whole-rocks
References

Sample description and data treatment

1257 A.D. samples

Glassy melt inclusions are present in most crystal phases of the 1257 A.D. pumice clasts (ortho-(opx) and clino-(cpx) pyroxene, amphibole and plagioclase), and are most abundant in plagioclase. These melt inclusions are squared or irregular in shape, with sizes ranging from $\sim$10 μm to $\sim$60 μm (Supplementary Figure S1). They are trapped in both calcic plagioclase cores and more sodic rims of zoned plagioclase (An_{49} to An_{86}), recording the complex history of the 1257 magma. Some melt inclusions bear sulfide globules of up to $\sim$4 μm in diameter. A total of 41 melt inclusions were analysed in plagioclase from pumice clasts of phases P1, P2 and P3 for major element and volatiles, among which 18 melts inclusions from P1 and P3 were analysed for trace element and 20 for H_2O and CO_2 by FT-IR spectroscopy. We analysed 9 melt inclusions in cpx, 15 melt inclusions in opx and 6 melt inclusions in amphibole for major elements, S and Cl. Matrix glasses of pumice clasts from P1, P2, P3 and P4 fallout units were analysed in 8 samples from proximal and distal outcrops[1]. Matrix glasses from pumices clasts erupted during phases P3 and P4 contain systematically microlites of feldspar, pyroxene and Fe-Ti oxide. Average compositions are reported in Supplementary Table S2.

712 A.D. samples

Glassy melt inclusions occur in the Fo_{71-77} olivine of the 712 A.D. scoria fallout (Métrich et al., submitted). Some olivine crystals contain inclusions that are partly or entirely crystallized. Melts inclusions are perfectly rounded with diameters ranging from $\sim$10 μm to $\sim$120 μm (Supplementary Figure S1). A total of 20 melt inclusions were analysed for major elements and volatiles, among which 16 melt inclusions were analysed for H_2O and CO_2 by FT-IR spectroscopy, and 14 for trace elements. The mineral/melt equilibirum was tested for all melt inclusions of the dataset. Given that the melt inclusion/olivine distribution coefficient ${K_D}_{ol-liq}^{Mg-Fe}$ depends on oxygen fugacity, ${K_D}_{ol-liq}^{Mg-Fe}$ values were calculated for NNO (K_D of 0.29-0.37) and NNO+0.5 (K_D of 0.30-0.39) at 1040 °C[2]. Low K_D values (0.29-0.35) are consistent with equilibrium between melt inclusions and their host olivine, thus excluding post entrapment crystallization processes. 6 melt inclusions displayed K_D values $\geq$0.35, and theoretical equilibrium olivine composition Fo_{th} $\geq$4% higher than the measured forsterite content $Fo_{measured}$ of their host, suggesting minor Fe-loss[3]. These melt inclusions were excluded from further consideration, except two of them that displayed acceptable K_D values (0.36) given the uncertainty on the redox state. Average compositions are reported in Supplementary Table S3.

2550 B.P. samples

Plagioclase crystals (An_{73} to An_{77}) from the 2550 B.P.[4] pumice fallout contain abundant glassy melt inclusions, most of which are irregular in shape. The most regular squarish melt inclusions are up to $\sim$50 μm in size. Plagioclase-hosted melt inclusions in the 1257 and 2550 B.P. samples are dominantly located in the large cores together with glass. The melt inclusions most suitable for analysis are distributed along the growth plans of their host mineral. A total of 13 melt inclusions were analysed for major elements and volatiles (including H_2O and CO_2 by FT-IR spectroscopy), among which 6 melt inclusions were analysed for trace element (Supplementary Table S3).

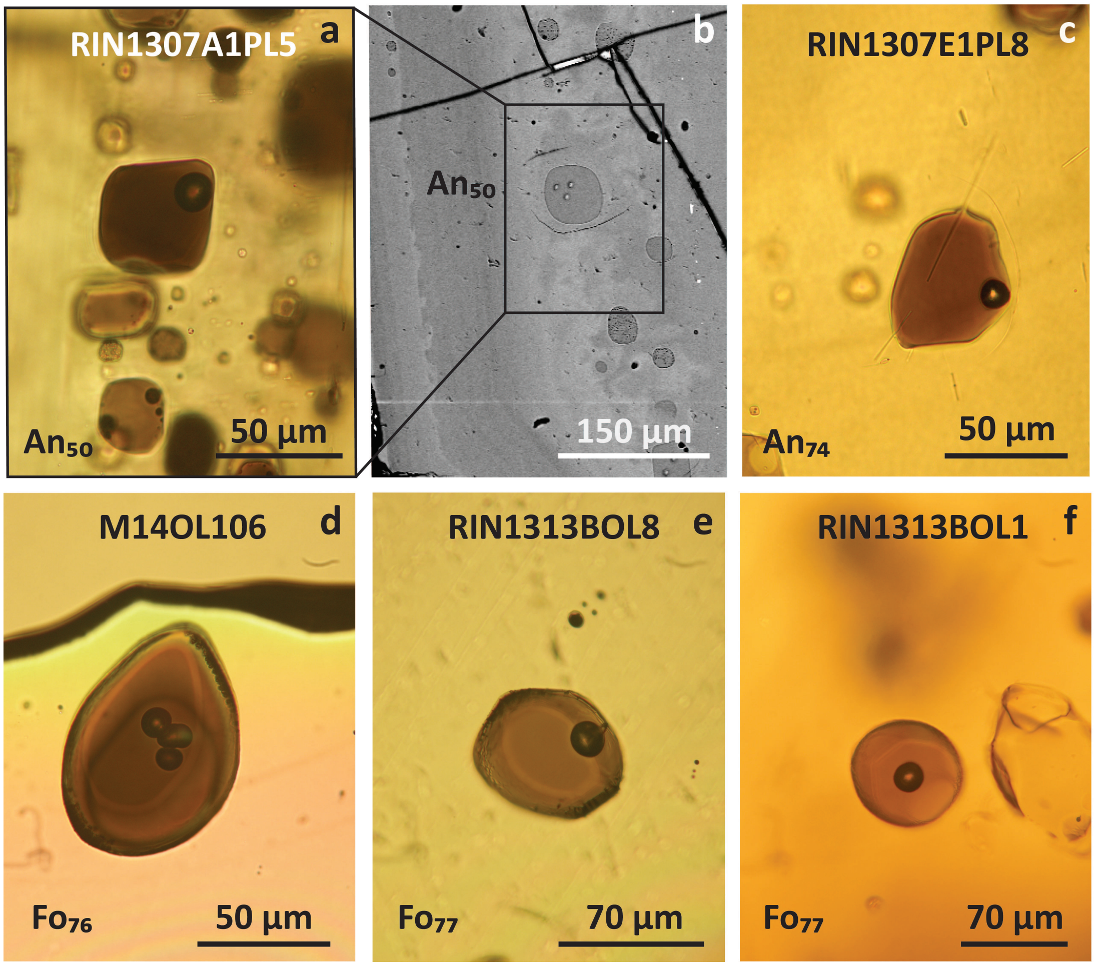

Figure S1. Representative microphotographs of melt inclusions in this study
a Transmitted light microphotograph before double polishing and **b** Backscattered electron scanning microscope (BSE) photographs of plagioclase-hosted melt inclusion RIN1307A1PL5 from phase P1 of the 1257 eruption. **c** Transmitted light of double-polished plagioclase-hosted melt inclusion RIN1307E1PL8 from phase P3 of the 1257 eruption. **d** Transmitted light microphotograph of doubled-polished olivine-hosted melt inclusion M14OL106 from 712 A.D. scoria. **e** and **f** Transmitted light microphotographs of doubled-polished olivine-hosted melt inclusions RIN1313BOL1 and RIN1313BOL8 from 712 A.D. scoria.

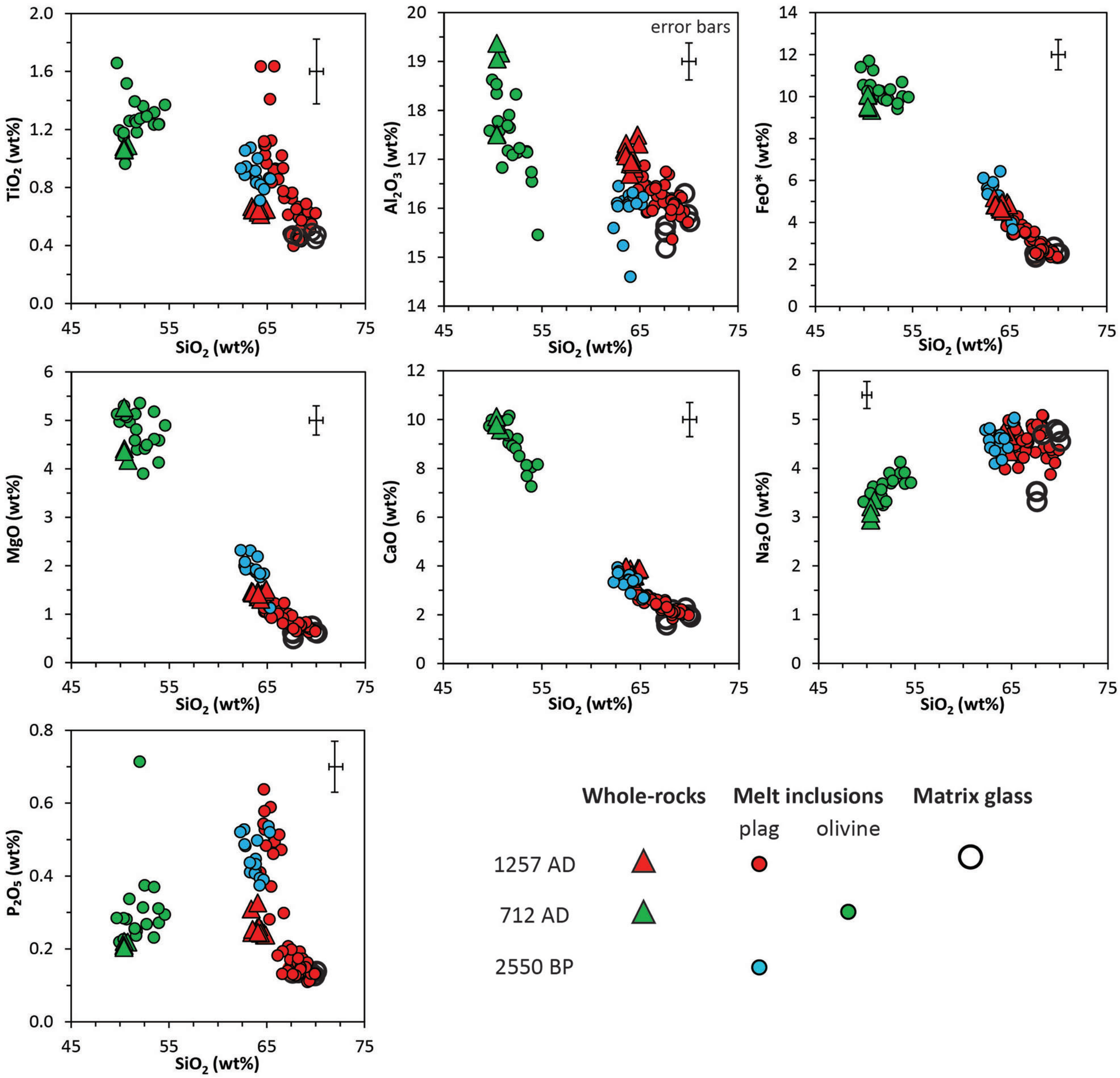

Figure S2. Major element variation diagrams of the Rinjani-Samalas magmas

Compositions of melt inclusions, whole-rocks and matrix glasses reflect the chemical bimodality of the Rinjani-Samalas magmas including the 712 A.D. basalts, the 2550 B.P. trachydacite and the 1257 A.D. trachydacite. All analyses are normalised to 100 wt%, free of volatiles.

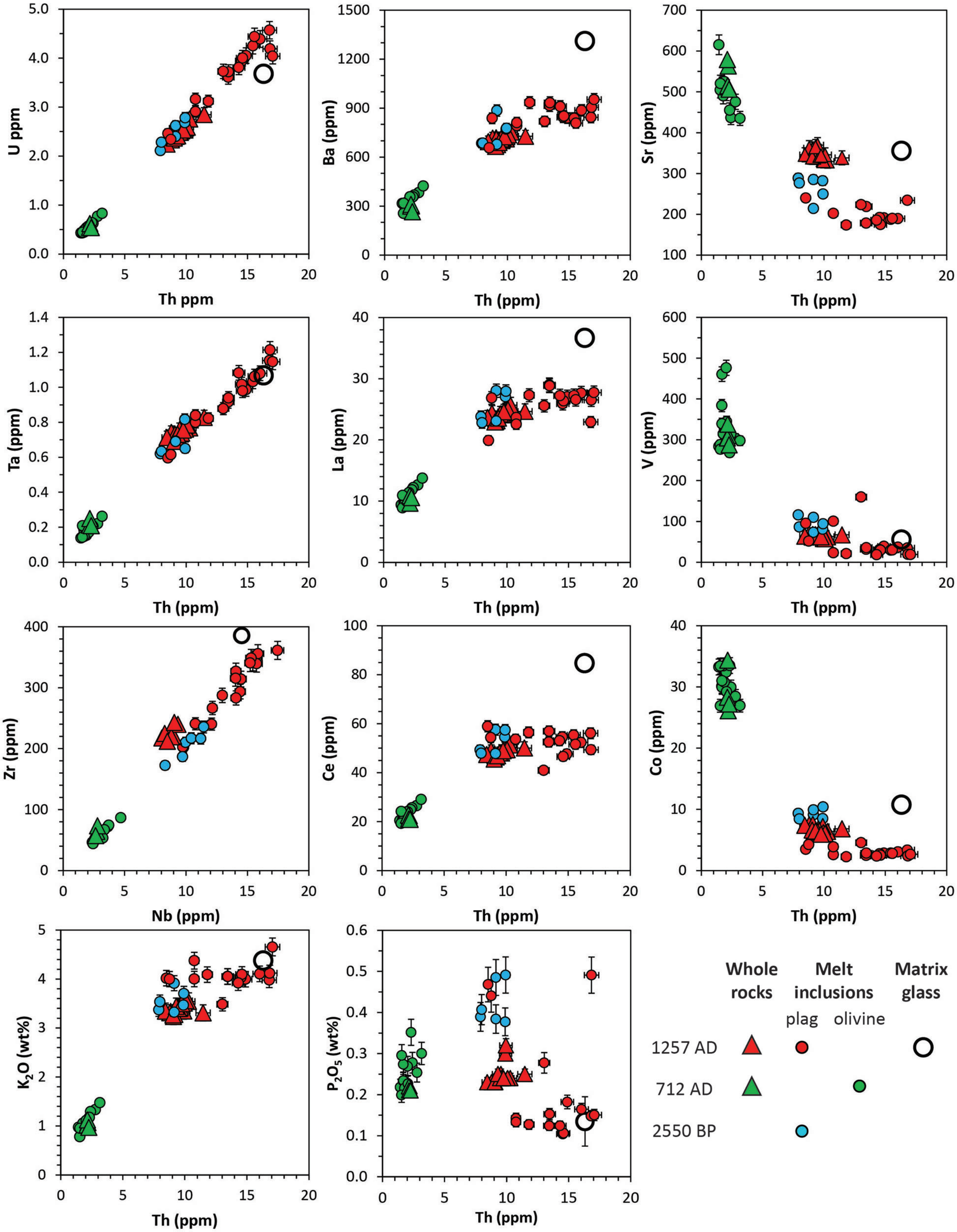

Figure S3. Trace element variation diagrams for the Rinjani-Samalas magmas

During in-situ crystallization (Th>10 ppm), K_2O, Ba, La and Ca do not behave as incompatible elements due to fractioning by amphibole and apatite. Error bars are reported for each point in each plot.

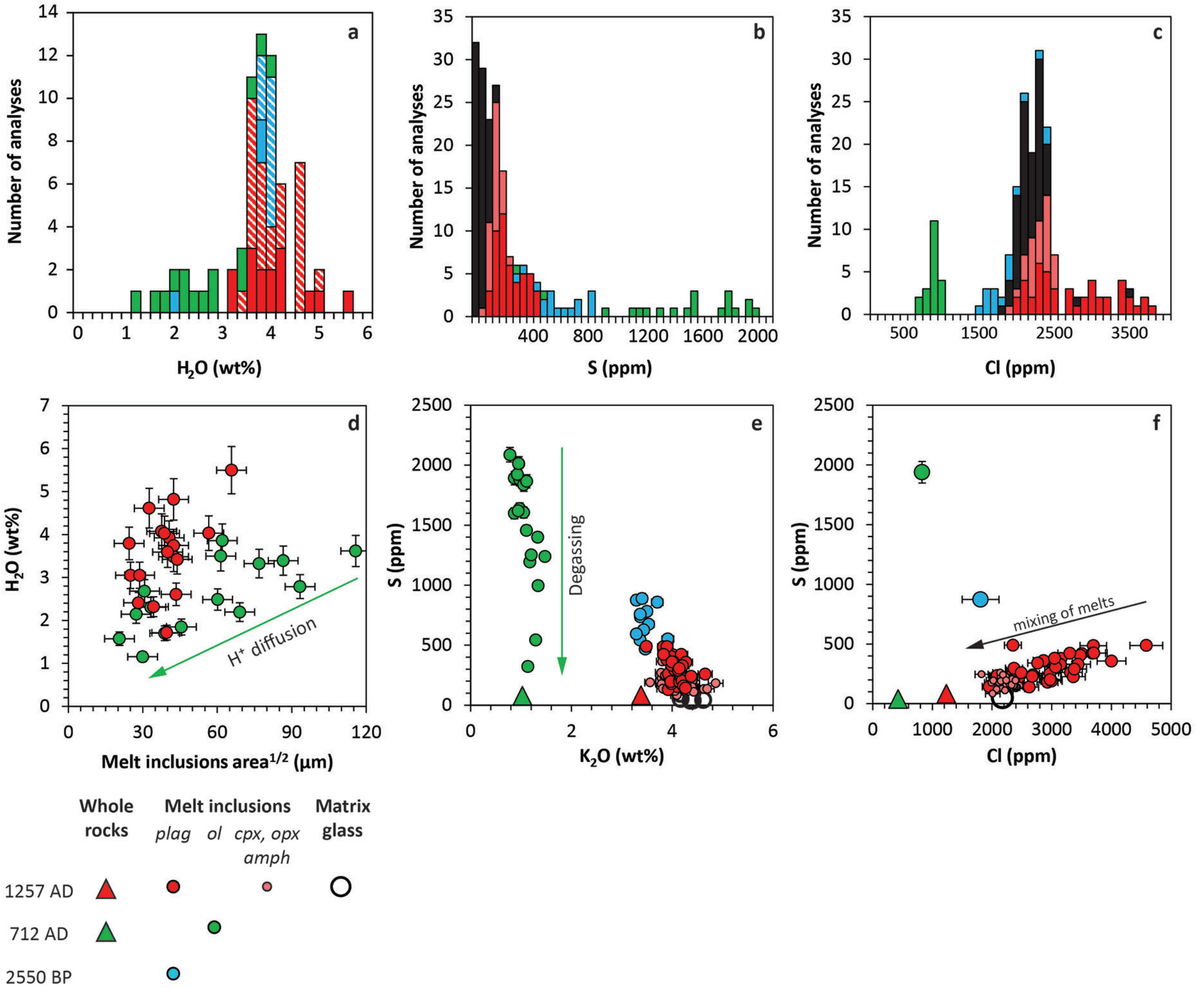

Figure S4. Volatile (H_2O, S and Cl) concentrations in melt inclusions and matrix glasses

a b and **c** Number of analyses in melt inclusions and matrix glasses for H_2O, S and Cl. Hatched histograms are water concentrations calculated with the plagioclase-liquid equilibrium hygrometer of Lange et al. (2009)[5] and the composition of the host plagioclase. **d** Dissolved H_2O content (FT-IR) in basaltic melt inclusions is positively correlated to their size suggesting proton diffusion trough host olivine network[6], whereas trachydacitic plagioclase-hosted melt inclusions display variable water contents independently from their size. **e** S vs K_2O contents in melt inclusions, matrix glasses and whole rocks showing the record of S degassing associated with the 712 AD eruption. **f** The positive correlation of S and Cl contents in trachydacitic melt inclusions reflects the processes of mixing of batches of melts displaying distinct volatile contents. Error bars are reported for each point in each plot.

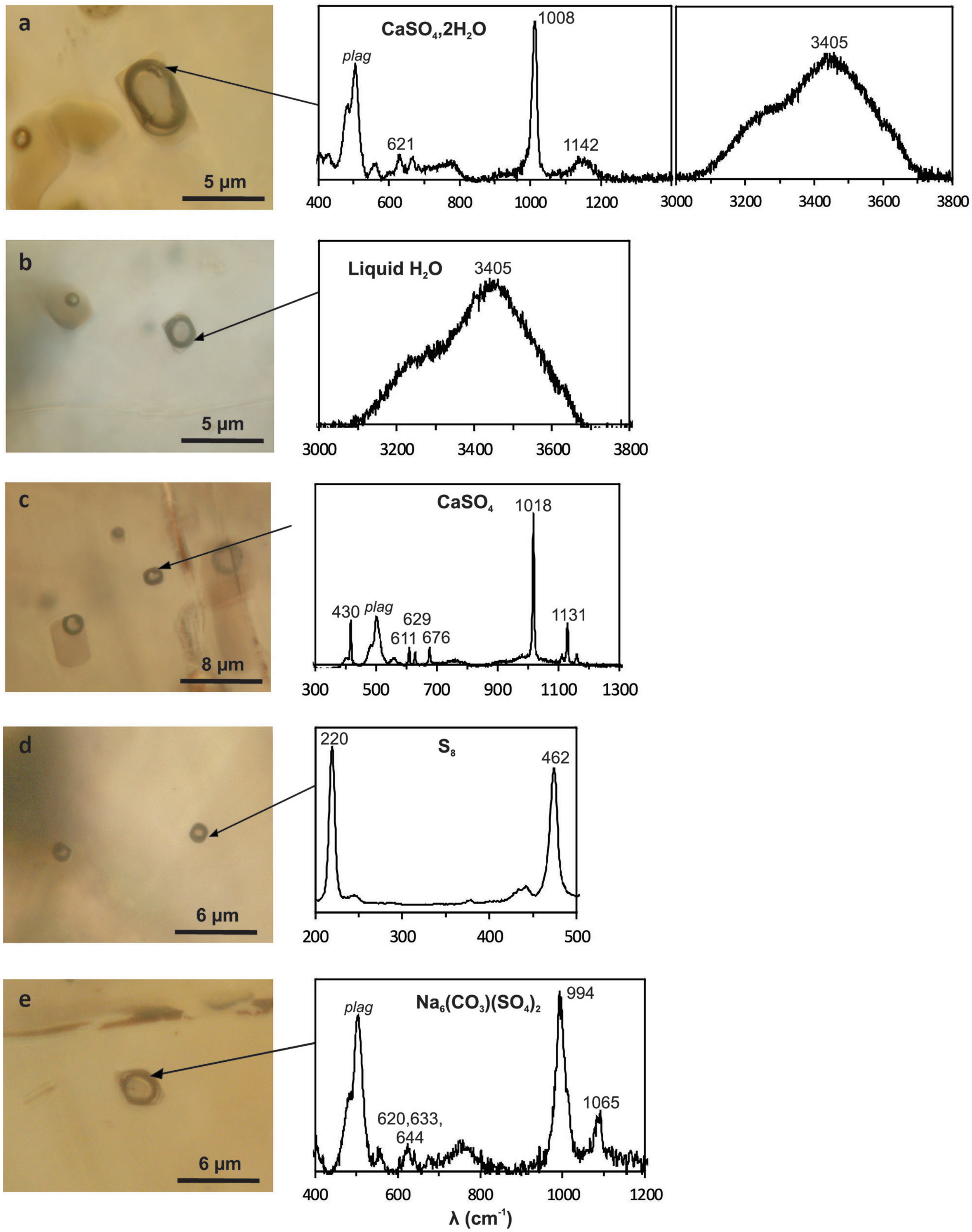

Figure S5. Plagioclase-hosted fluid inclusions in the 1257 eruptive products
Associated Raman spectras witness the presence of sulphur, H_2O and S in the pre-existing vapour phase.

Table 1. Average whole-role major element (ICP-AES), sulphur and halogens (pyrohydrolysis/ICP-MS) and trace element (ICP-MS) data for 1257 A.D. and pre-1257 samples

Sample	**1257 A.D.**		**712 A.D.**	
n[1]	*9*	SD[2]	*3*	SD
SiO_2 *(wt%)*	62.3	0.7	50.3	0.8
TiO_2	0.63	0.01	1.06	0.03
Al_2O_3	16.6	0.3	19.1	0.2
Fe_2O_{3Total}	4.7	0.1	10.43	0.06
MnO	0.14	<0.01	0.19	<0.01
MgO	1.39	0.06	4.29	0.11
CaO	3.65	0.18	9.70	0.16
Na_2O	4.09	0.68	3.20	0.18
K_2O	3.38	0.08	1.02	0.05
P_2O_5	0.24	0.01	0.21	0.01
Total	97.1		99.5	
SiO_2 Norm[3]	64.2	0.5	50.5	0.2
(Na_2O+K_2O) Norm	7.7	0.7	4.2	0.2
S *(ppm)*	83	23	39	1
Cl	1234	45	428	0
F	560	14	228	6
Br *(ppb)*	2916	78	1595	178
As	3.6	0.3	n.a.[3]	n.a.
Ba	707	19	288	20
Be	1.5	0.1	0.7	0.0
Cd	0.26	0.03	0.16	0.03
Ce	48	1	22	1
Cs	3.0	0.2	0.6	0.1
Co	6.7	0.5	27.2	1.1
Cu	13	4	95	9
Dy	4.6	0.2	3.4	0.1
Er	2.8	0.1	1.9	0.0
Eu	1.27	0.04	1.18	0.03
Ga	17.7	0.3	20.2	0.4
Gd	4.6	0.1	3.35	0.08
Ge	1.50	0.04	1.55	0.07
Hf	5.7	0.2	1.8	0.1
Ho	0.98	0.06	0.70	0.02
La	24.1	0.6	10.7	0.2
Lu	0.50	0.01	0.31	0.01
Mo	3.2	0.2	0.91	0.08
Nb	8.7	0.4	2.74	0.08
Nd	22.5	0.4	12.7	0.4
Pb	14	1	5.3	0.3
Pr	5.7	0.1	2.86	0.06
Rb	83	3	19	1
Sc	9.7	0.5	30.4	0.3
Sb	0.29	0.03	3.30	0.07
Sm	5.0	0.1	0.8	0.1
Sr	351	12	549	38
Ta	0.75	0.04	0.212	0.004
Tb	0.74	0.01	0.54	0.01
Th	9.5	0.9	2.20	0.07
Tm	0.444	0.009	0.289	0.006
U	2.5	0.2	0.55	0.01
V	65	3	301	12
Y	28.8	0.5	19.8	0.5
Yb	3.12	0.07	1.95	0.03
Zr	224.7	10.2	64.9	8.2
Rb/Th	8.8	0.7	8.5	0.4
Zr/Nb	26	1	24	2
Br/Cl	0.0022	0.0003	0.0037	0.0004

[1] n: number of averaged values

[2] SD is the standard deviation of the mean (1σ)

[3] Normalized on anhydrous basis

[4] n.d., not detected; n.a., not analysed

Table 2. Average compositions of matrix glasses and melt inclusions trapped in plagioclase, cpx, opx and amphibole from the 1257 pumices

Mineral phase	Plagioclase	An_{50-57}	Plagioclase	An_{72-81}	Clinopyroxene		Orthopyroxene		Amphibole		Matrix glass	
n[1]	*15*	SD[2]	*3*	SD	*9*	SD	*15*	SD	*6*	SD	*94*	SD
SiO_2 *(wt%)*	64.8	0.1	61.7	0.1	66.1	0.9	65.6	1.4	64.9	0.7	68.7	1.1
TiO_2	0.6	0.2	0.9	0.1	0.46	0.06	0.47	0.06	0.47	0.05	0.47	0.01
Al_2O_3	15.1	0.3	15.3	0.4	15.2	0.5	15.4	0.4	15.6	0.3	15.8	0.3
FeO_{Total}	2.61	0.05	3.79	0.06	2.59	0.09	3.0	0.1	2.7	0.2	2.6	0.1
MnO	0.1	0.1	0.2	0.1	0.08	0.02	0.13	0.04	0.10	0.04	0.12	0.01
MgO	0.7	0.2	1.0	0.1	0.58	0.07	0.64	0.05	0.65	0.05	0.63	0.08
CaO	2.1	0.2	2.6	0.4	1.9	0.1	2.1	0.2	2.1	0.2	1.9	0.2
Na_2O	4.1	0.2	4.4	0.1	4.5	0.3	4.6	0.2	4.67	0.07	4.2	0.7
K_2O	4.00	0.05	3.94	0.09	4.3	0.2	4.1	0.4	4.2	0.2	4.4	0.2
P_2O_5	0.1	0.5	0.5	0.8	0.2	0.1	0.12	0.01	0.131	0.009	0.13	0.06
H_2O	3.9	0.6	3.7	1.2	n.a[3]	n.a.	n.a	n.a.	n.a	n.a.		
Total	98.1		98.1		95.9		96.3		95.4		98.8	
S *(ppm)*	236	78	348	90	150	20	180	46	183	45	51	22
Cl	2429	390	3377	455	2282	139	2232	139	2172	211	2171	109
Sc	10.5	1.5	17.7	4.0								
V	31	10	118	36							55*	
Co	2.8	0.6	4.0	0.5							10.8	
Ni	n.d.	n.d.	n.d.	n.d.							n.d.	
Cu	6.5	1.0	12.7	5.5							n.d.	
Rb	123	16	108	12							118	
Sr	196	20	226	28							356	
Y	31.9	3.6	35.8	7.7							36.1	
Zr	303	47	242	41							396	
Nb	13.9	2.2	12.0	2.1							14.5	
Ba	875	45	762	92							1311	
La	26.6	1.8	22.7	2.8							36.7	
Ce	53.4	3.3	47.7	6.8							84.7	
Pr	6.0	0.4	5.7	0.9							8.5	
Nd	25.0	1.7	25.1	3.8							32.9	
Sm	5.5	0.6	6.4	0.8							6.8	
Eu	1.1	0.1	1.0	0.1							1.7	
Gd	5.0	0.6	6.2	1.5							6.8	
Tb	0.8	0.1	0.9	0.2							1.07	
Dy	5.4	0.6	6.2	1.8							6.6	
Ho	1.1	0.1	1.3	0.3							1.4	
Er	3.5	0.4	3.9	1.0							4.3	
Tm	0.6	0.1	0.6	0.2							0.71	
Yb	4.0	0.5	4.2	1.3							4.6	
Lu	0.6	0.1	0.6	0.2							0.76	
Hf	7.6	1.2	5.9	1.3							10.0	
Ta	1.0	0.2	0.8	0.2							1.07	
Pb	19	2	19	5							20.1	
Th	13.9	2.4	10.8	2.3							16.3	
U	3.7	0.6	3.1	0.6							3.7	

[1] n: number of averaged values
[2] SD is the standard deviation of the mean (1σ)
[3] n.d., not detected; n.a., not analysed
* Trace element compositions of pumice clasts $<63\mu m$ from Métrich et al. (submitted)

Table 3. Average compositions of 2550 B.P. plagioclase-hosted melt inclusions representative of the 1257 A.D. whole-rock and 712 A.D. olivine-hosted melt inclusions representative of the 712 A.D. basaltic scoria

Sample	**2550 B.P.**				**712 A.D.**			
Mineral phase	Plagioclase				Olivine			
n[1]	*6*	SD[2]	Min	Max	*8*	SD	Min	Max
SiO_2 *(wt%)*	60.5	1.3	58.8	62.4	48.5	0.6	47.8	51.6
TiO_2	0.85	0.07	0.66	1.00	1.1	0.1	0.9	1.5
Al_2O_3	15.0	0.5	14.2	15.7	17.1	0.7	14.6	17.9
FeO_{Total}	5.2	0.7	4.5	6.3	9.8	0.6	8.9	11.2
MnO	0.15	0.02	0.06	0.19	0.20	0.02	0.18	0.26
MgO	1.9	0.2	1.7	2.2	4.6	0.5	3.7	5.1
CaO	3.2	0.2	2.8	3.7	9.2	0.5	7.7	9.6
Na_2O	4.3	0.2	3.9	4.6	3.2	0.3	2.8	3.7
K_2O	3.6	0.2	3.3	3.9	1.0	0.1	0.8	1.3
P_2O_5	0.42	0.05	0.35	0.50	0.24	0.03	0.20	0.35
H_2O	3.9	0.1	1.6	3.7	3.5	0.2	1.2	3.9
Total	98.9				98.5			
CO_2 *(ppm)*	n.d.[3]	n.d.	n.d.	n.d.	508	298	270	826
S	647	139	469	888	1939	88	323	2088
Cl	1807	309	1455	2310	827	77	668	994
Sc	15.1	2.0	13	18	28.8	2.0	24	31
V	93	17	73	116	343	58	268	476
Co	9.3	0.8	8	10	30.6	2.1	27	33
Ni	n.d.	n.d.	n.d.	n.d.	16.4	5.1	10.0	29.8
Cu	11.4	1.8	9	14	114	32	54	248
Rb	84	9	71	95	18	2	14	27
Sr	266	29	214	289	512	11	437	615
Y	32.2	4.4	28	39	18.6	1.5	16	22
Zr	206	23	173	236	52	4	44	74
Nb	10.2	1.1	8.3	11.4	2.8	0.3	2.4	3.7
Ba	747	81	679	885	303	30	256	367
La	25.5	2.5	23	28	10.0	0.8	9	12
Ce	52.4	4.6	48	58	21.4	1.8	19	26
Pr	6.1	0.6	5.5	6.9	2.7	0.2	2.4	3.1
Nd	26.6	3.1	24	31	12.9	1.2	11	15
Sm	5.9	0.7	4.9	6.6	3.4	0.3	3.1	4.0
Eu	1.5	0.2	1.3	1.7	1.1	0.1	1.0	1.4
Gd	5.7	1.1	4.6	7.6	3.3	0.3	2.9	3.7
Tb	0.9	0.1	0.8	1.1	0.53	0.04	0.5	0.6
Dy	5.8	0.8	4.9	7.2	3.5	0.2	3.1	4.4
Ho	1.1	0.1	1.0	1.3	0.7	0.1	0.6	0.8
Er	3.4	0.5	2.8	4.2	2.1	0.2	1.9	2.4
Tm	0.5	0.1	0.4	0.6	0.3	0.0	0.2	0.4
Yb	3.5	0.4	3.0	4.1	2.1	0.3	1.6	2.7
Lu	0.5	0.1	0.4	0.6	0.31	0.05	0.2	0.4
Hf	5.1	0.6	4.3	5.7	1.5	0.1	1.3	2.0
Ta	0.7	0.1	0.6	0.8	0.17	0.02	0.1	0.2
Pb	13.7	2.1	12	18	5.6	0.6	5	9
Th	9.0	0.9	7.9	9.9	1.7	0.2	1.4	2.4
U	2.5	0.3	2.1	2.8	0.48	0.05	0.4	0.6

[1] n: number of averaged values
[2] SD is the standard deviation of the mean (1σ)
[3] n.d., not detected; n.a., not analysed

Table 4. Average composition (EPMA) of sulphides in phenocrysts of the 1257 A.D., 712 A.D. and 2550 B.P. eruptive products

Sample	712 A.D.		2550 B.P.		1257 A.D.	
Sulphide	Cu-Fe-S		Fe-S		Fe-S	
n[1]	*3*	SD[2]	*5*	SD	*2*	SD (wt%)
S *(wt%)*	32.1	0.6	38.6	0.4	38.9	0.5
Fe	39.1	1.9	57.5	1.3	58.4	0.1
Ni	0.4	0.2	0.1	0.1	n.d.[3]	n.d.
Cu	27.0	1.1	1.7	0.4	1.3	0.2
Co	0.03	0.03	0.04	0.03	0.07	0.02
Mn	0.07	0.02	0.11	0.03	0.06	0.01
Si	0.07	0.09	0.008	0.007	0.018	0.005
Total	98.92		97.99		98.65	

[1] n: number of averaged values
[2] SD is the standard deviation of the mean (1σ)
[3] n.d. not detected

Table 5. Average composition (EPMA) of apatite in phenocrysts of the 1257 A.D. samples (4 phases)

	**n[1]=168**	**SD[2]**	**Min**	**Max**
CaO *wt%*	54.2	0.6	50.93	55.13
FeO	0.6	0.1	0.36	1.56
P_2O_5	42.8	0.7	39.66	43.96
F	1.55	0.07	1.41	1.77
S	0.07	0.05	0.01	0.26
Cl	0.89	0.02	0.81	0.95

[1] n: number of averaged values
[2] SD is the standard deviation of the mean (1σ)

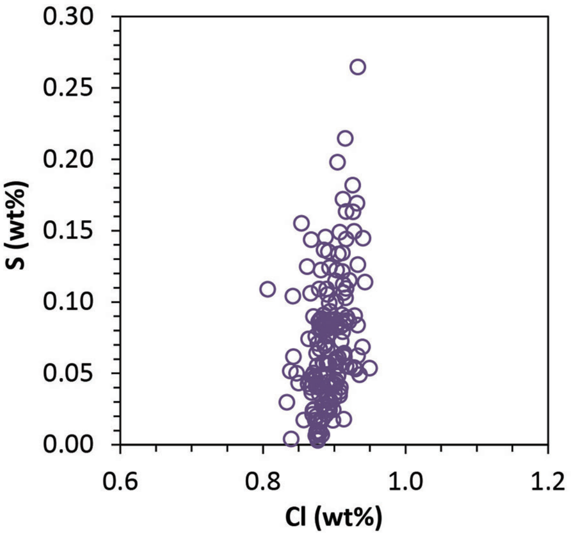

Figure S6. Sulphur and chlorine composition of apatite from the 1257 A.D. eruptive products
Analyses from the four phases of the eruption are reported.

Table 6. Average composition (EPMA) of amphibole in the 1257 A.D. samples (4 phases)

	n[1]=265	SD[2]	Min	Max
SiO_2 *wt%*	43.22	0.64	40.32	44.98
TiO_2	3.36	0.24	2.74	3.84
Al_2O_3	10.68	0.54	9.86	13.09
FeO	12.42	0.23	11.77	13.35
MnO	0.38	0.04	0.20	0.50
MgO	13.85	0.27	12.91	14.61
CaO	11.26	0.14	10.70	11.66
Na_2O	2.42	0.08	2.21	2.69
K_2O	0.78	0.06	0.57	0.92
F	0.25	0.03	0.17	0.33
Cl	0.07	0.01	0.02	0.10
Total	98.69			

[1] n: number of averaged values

[2] SD is the standard deviation of the mean (1σ)

Table 7. Average trace element concentrations of international standard BCR-2G by LA-ICP-MS compared to published data

	This work			Sigmarsson et al. (2013)[7]		Jenner et al. (2012)[8]			Jochum et al. (2014)[9]		
n[1]	*10*	SD[2]	RSD[3](%)	*57-136*	2RSE (%)	*14-259*	SD	RSD (%)	*12*	SD	RSD
Sc *(ppm)*	33.50	1.20	3.6	32.96	0.69	34.40	0.30	0.9	33*	2.00	6.1
V	432.78	3.79	0.9	433.69	-	436.00	5.00	1.1	422.00	11.14	2.6
Co	38.76	0.83	2.2	38.22	-	39.70	0.50	1.2	36.80	0.92	2.5
Ni	12.67	0.67	5.3	12.39	-	12.60	0.20	1.6	14.57	2.12	14.6
Cu	18.17	0.69	3.8	18.25	1.38	19.60	0.40	2.1	17.70	1.35	7.6
Rb	49.08	1.39	2.8	49.55	0.89	48.70	0.60	1.3	46.13	1.50	3.3
Sr	334.56	6.06	1.8	330.10	0.38	339.00	5.00	1.3	329.67	4.04	1.2
Y	31.42	1.32	4.2	30.54	0.74	32.30	0.20	0.8	31.73	1.42	4.5
Zr	169.57	7.01	4.1	164.80	0.79	181.00	7.00	3.9	172.67	7.77	4.5
Nb	12.29	0.35	2.9	12.14	0.61	12.70	0.30	2.4	11.90	0.46	3.9
Ba	675.93	8.65	1.3	674.25	0.78	706.00	7.00	1.1	664.33	16.77	2.5
La	24.20	0.60	2.5	23.84	0.49	24.50	0.70	2.9	24.03	0.72	3.0
Ce	51.87	0.68	1.3	52.00	0.57	51.10	0.60	1.2	50.77	1.66	3.3
Pr	6.41	0.11	1.7	6.36	0.61	6.46	0.12	1.8	6.38	0.12	1.9
Nd	27.96	0.79	2.8	27.42	0.72	27.50	0.30	1.3	27.30	0.56	2.0
Sm	6.41	0.25	3.9	6.28	0.97	6.41	0.10	1.5	6.39	0.18	2.8
Eu	1.86	0.10	5.1	1.85	0.94	1.98	0.04	2.1	1.87	0.05	2.6
Gd	6.11	0.37	6.0	6.01	1.09	6.29	0.11	1.8	6.24	0.26	4.1
Tb	0.94	0.06	6.1	0.90	1.03	0.97	0.02	1.8	0.93	0.06	6.7
Dy	6.13	0.28	4.5	5.87	0.89	6.08	0.12	2.0	5.93	0.25	4.2
Ho	1.20	0.06	5.2	1.16	0.97	1.24	0.02	2.0	1.18	0.06	5.0
Er	3.40	0.14	4.2	3.30	1.04	3.46	0.04	1.1	3.40	0.19	5.7
Tm	0.47	0.03	6.5	0.47	1.70	0.48	0.01	1.7	0.46	0.03	5.8
Yb	3.25	0.20	6.2	3.14	1.27	3.42	0.08	2.4	3.11	0.10	3.2
Lu	0.48	0.03	6.8	0.45	1.54	0.49	0.02	3.4	0.46	0.03	5.4
Hf	4.38	0.25	5.8	4.21	1.06	4.76	0.20	4.3	4.57	0.12	2.6
Ta	0.76	0.05	6.1	0.74	1.36	0.73	0.01	1.4	0.70	0.03	4.3
Pb	10.94	0.25	2.3	10.94	1.24	10.90	0.20	1.4	10.63	0.83	7.8
Th	5.65	0.19	3.4	5.45	0.84	5.82	0.27	4.6	5.62	0.23	4.1
U	1.73	0.07	3.9	1.73	1.30	1.66	0.03	1.7	1.65	0.12	7.3

[1] n: number of averaged values
[2] SD is the standard deviation of the mean (1σ)
[3] RSD is the relative standard deviation (100' s/mean)
* Data from Jochum et al. (2008)[10]

Table 8. Sulphur and halogen composition of standards SY-2 (syenite), BHVO-1 (basalt), BE-N (basalt), AGV-1 (andesite) and JR-1 (rhyolite) extracted by pyrohydrolysis

	This work	RSD (%) [2]	G.1994 & I. 1995[3]	GeoReM >2000[4]	M & V 2003[5]
Sulphide bearing syenite SY2					
n[1]	*4*				
S *(ppm)*	116	11	160	121.5±5.6	115± 25
Basalt BHVO-1					
n	*8*				
F *(ppm)*	394	12.8	385± 31 R	413± 4	
Cl *(ppm)*	79	6.8	92 R	90±7	
Br *(ppm)*	0.25	15.6	n.a.	0.22±0.07[*]	
S *(ppm)*	90	17.3	102 R	93± 6; 54±5	
Anorthosite AGV-1					
n	*5*				
F *(ppm)*	450	5.9	425± 50	402± 12	
Cl *(ppm)*	102	4.0	119±26	170±3; 107±10	
Br *(ppm)*	0.29	10.8	0.32	0.34±0.01[*]	
Basalt BE-N					
n	*4*				
F *(ppm)*	1077	0.7	1000±152[R]		1080±7
Cl *(ppm)*	170	10	200±88		179±8
Br *(ppm)*	0.34	6.0		0.32±0.05[*]	0.6±0.1[*]
S *(ppm)*	298	2.3	300±150	312±14[*]	308±31
Rhyolite JR-1					
n	*3*				
F *(ppm)*	1011	2.7	991[R]	1034±40; 1011±28	
Cl *(ppm)*	913	1.0	920[R]	951±67	
Br *(ppm)*	0.30	11.5		2.1±0.2[*]	

[1] n: number of average values
[2] RSD is the relative standard deviation (100’ s/mean)
[3] SY2, BHVO-1, AGV-1, BE-N: Govindaraju (1994)[11]; JR-1: Imai et al. (1995)[12]
[4] Average values from GeoREM database of the last 15 years with associated RSD. GeoREM: http://georem.mpch-mainz.gwdg.de/
[5] Michel and Villemant (2003)[13]
[R] Reference value
[*] One measurement

References

1. Vidal, C. M. *et al.* Dynamics of the major plinian eruption of Samalas in 1257 A.D. (Lombok, Indonesia). *Bull. Volcanol.* **77**, 73 (2015).
2. Foden, J. D. & Green, D. H. Possible role of amphibole in the origin of andesite: some experimental and natural evidence. *Contrib. to Mineral. Petrol.* **109**, 479–493 (1992).
3. Danyushevsky, L. V., Della, F. N. & Sokolov, S. Re-equilibration of melt inclusions trapped by magnesian olivine phenocrysts from subduction-related magmas : petrological implications. *Contrib. to Mineral. Petrol.* **138**, 68–83 (2000).
4. Nasution, A., Takada, A. & Mulyana, R. The volcanic activity of Rinjani, Lombok island, Indonesia, during the last 10000 years, viewed from 14C ages (Indonesian Association of Geologist, Convention Bandung 2004, 2004).
5. Lange, R. a., Frey, H. M. & Hector, J. A thermodynamic model for the plagioclase-liquid hygrometer/thermometer. *Am. Mineral.* **94**, 494–506 (2009).
6. Gaetani, G. A., O'Leary, J. A., Shimizu, N., Bucholz, C. E. & Newville, M. Rapid reequilibration of H2O and oxygen fugacity in olivine-hosted melt inclusions. *Geology* **40**, 915–918 (2012).
7. Sigmarsson, O. *et al.* Formation of U-depleted rhyolite from a basanite at El Hierro, Canary Islands. *Contrib. to Mineral. Petrol.* **165**, 601–622 (2013).
8. Jenner, F. E. & O'Neill, H. S. C. Major and trace analysis of basaltic glasses by laser-ablation ICP-MS. *Geochemistry, Geophys. Geosystems* **13**, 1–17 (2012).
9. Jochum, K. P. *et al.* Non-Matrix-Matched Calibration for the Multi-Element Analysis of Geological and Environmental Samples Using 200 nm Femtosecond LA-ICP-MS: A Comparison with Nanosecond Lasers. *Geostand. Geoanalytical Res.* **38**, 265–292 (2014).
10. Jochum, K. P. & Nohl, U. Reference materials in geochemistry and environmental research and the GeoReM database. *Chem. Geol.* **253**, 50–53 (2008).
11. Govindaraju, K. Compilation of working values and sample descriptions for 383 geostandards. *Geostand. Newletter* **18** (1994).
12. Imai, N. & TERASHIMA, S. 1994 compilation of analytical data for minor and trace elements in seventeen GSJ geochemical reference samples,"igneous rock series". *Geostand. ...* (1995).
13. Michel, A. & Villemant, B. Determination of halogens (F, Cl, Br, I), sulfur and water in seventeen geological reference materials. *Geostand. Geoanalytical Res.* **27**, 163–171 (2003).
